# The expression of *Spodoptera exigua* P450 and UGT genes: tissue specificity and response to insecticides

**DOI:** 10.1111/1744-7917.12538

**Published:** 2017-11-21

**Authors:** Bo Hu, Shu‐Heng Zhang, Miao‐Miao Ren, Xiang‐Rui Tian, Qi Wei, David Kibe Mburu, Jian‐Ya Su

**Affiliations:** ^1^ Key Laboratory of Integrated Management of Crop Diseases and Pests (Ministry of Education) College of Plant Protection Nanjing Agricultural University Nanjing China

**Keywords:** abamectin, cytochrome P450 monooxygenases, gene expression, insecticide, *Spodoptera exigua*, UDP‐glycosyltransferase

## Abstract

Cytochrome P450 and UDP‐glucosyltransferase (UGT) as phase I and phase II metabolism enzymes, respectively, play vital roles in the breakdown of endobiotics and xenobiotics. Insects can increase the expression of detoxification enzymes to cope with the stress from xenobiotics including insecticides. However, the molecular mechanisms for insecticide detoxification in *Spodoptera exigua* remain elusive, and the genes conferring insecticide metabolisms in this species are less well reported. In this study, 68 P450 and 32 UGT genes were identified. Phylogenetic analysis showed gene expansions in CYP3 and CYP4 clans of P450 genes and UGT33 family of this pest. P450 and UGT genes exhibited specific tissue expression patterns. Insecticide treatments in fat body cells of *S. exigua* revealed that the expression levels of P450 and UGT genes were significantly influenced by challenges of abamectin, lambda‐cyhalothrin, chlorantraniliprole, metaflumizone and indoxacarb. Multiple genes for detoxification were affected in expression levels after insecticide exposures. The results demonstrated that lambda‐cyhalothrin, chlorantraniliprole, metaflumizone and indoxacarb induced similar responses in the expression of P450 and UGT genes in fat body cells; eight P450 genes and four UGT genes were co‐up‐regulated significantly, and no or only a few CYP/UGT genes were down‐regulated significantly by these four insecticides. However, abamectin triggered a distinct response for P450 and UGT gene expression; more P450 and UGT genes were down‐regulated by abamectin than by the other four compounds. In conclusion, P450 and UGT genes from *S. exigua* were identified, and different responses to abamectin suggest a different mechanism for insecticide detoxification.

## Introduction

In response to the continuous exposure to xenobiotics including insecticides, insects have evolved an elaborate three‐phase detoxification system to protect themselves from possible toxicity from these foreign chemicals. The detoxification system of insects is mainly composed of biotransforming, metabolizing and excreting toxic xenobiotics (Li *et al*., 2007). The phase I detoxification enzymes consist of cytochrome P450 monooxygenases (P450s), esterases and flavin monooxygenases, which decrease the biological activity of a broad range of xenobiotics. The phase II enzymes, including glutathione S‐transferases (GSTs), UDP‐glucuronosyltransferases (UGTs), and sulfotransferase, act on the toxic by‐products of the phase I metabolism. The phase III transporters export the conjugated toxins out of the cell and include adenosine triphosphate‐binding cassette (ABC) and other transmembrane transporters (Tijet *et al*., 2001; Dermauw *et al*., 2013a; Liu *et al*., 2015; Bock, 2016).

The expression of detoxification genes can adapt to the needs for detoxification. It has been well established that the expression of these enzymes is regulated or induced by xenobiotic compounds in mammals (Abass *et al*., 2012). The induced expression of these enzymes in insects has been documented in recent years. P450s of *Manduca sexta* larvae can be induced to breakdown nicotine from tobacco leaves, and lead to increased tolerance to this noxious compound (Stevens *et al*., 2000). Cytochrome P (CYP)6B8 and CYP321A1 of *Helicoverpa zea* can be induced to metabolize several allelochemicals and insecticides (Li *et al*., 2004; Sasabe *et al*., 2004). Diet supplemented with xanthotoxin dramatically increases the level of CYP6B48 transcript in the midgut and fat body of *Spodoptera litura* (Wang *et al*., 2015). In *Bombyx mori*, the transcription level of some genes belonging to clan3 and clan4 CYP families are significantly up‐regulated after phoxim exposure (Li *et al*., 2015). Chlorpyrifos is responsible for the induced expression of GSTs in *S. litura* larvae (Zhang *et al*., 2016). In *Spodoptera frugiperda*, CYP450s from CYP6B, CYP321A and CYP9A subfamily can be induced by plant secondary metabolites such as indole, indole 3‐carbinol, 2‐tridecanone, xanthotoxin and so on. (Giraudo *et al*., 2015).

The up‐regulation of detoxification enzymes is of particular importance as it can further lead to insecticide resistance (Brun‐Barale *et al*., 2010; Dermauw *et al*., 2013b). The induced expression and constitutive overexpression of detoxification enzymes are thought to be responsible for enhanced levels of insecticide breakdown. The overexpression of CYP6BQ9 in *Tribolium castaneum* brain tissue resulted in the majority of deltamethrin resistances (Zhu *et al*., 2010), and overexpression of CYP6G1 in *Drosophila melanogaster*, caused resistance to DDT and imidacloprid (Daborn *et al*., 2001). Similarly, overexpression of CYP6Z1 in *Anopheles gambiae* is responsible for dithiothreitol resistance (Chiu *et al*., 2008).

The mechanisms for induced expression in mammals have been studied extensively, and transcription factors including aryl hydrocarbon receptor (AhR), pregnane X receptor (PXR), constitutive androstane receptor (CAR), and NF‐E2‐related factor 2 (Nrf2) have been shown to be the key mediators of xenobiotic‐induced changes in detoxification gene expressions (Xu *et al*., 2005). In insects the expression of xenobiotic metabolic genes are regulated by similar transcription factors. AhR are reported to mediate the response to xenobiotics and enhance the expression of CYP6B1 in *Papilio polyxenes* (Brown *et al*., 2005) and the expression of CYP6DA2 in *Aphis gossypii* (Peng *et al*., 2017). PXR ortholog HR96 modulates phenobarbital induced transcription of CYP6D1 in *Drosophila* S2 cells (Lin *et al*., 2011) and is required for the enhanced xenobiotic resistance in fruitfly (King‐Jones *et al*., 2006; Afschar *et al*., 2016). Nrf2 ortholog CncC is a central regulator of xenobiotic detoxification responses and contributes to the widespread overexpression of detoxification genes in insecticide resistant strains of *Drosophila* (Misra *et al*., 2011, 2013; Wan *et al*., 2014). The expression of CYP6DA2 in *A. gossypii* (Peng *et al*., 2016) and CYP6BQ in *T. castaneum* (Kalsi & Palli, 2015) are also regulated by CncC. Other regulatory factors, such as epidermal growth factor receptor (EGFR) is found to be involved in the increased expression of P‐glycoprotein in *Drosophila* (Luo *et al*., 2013), and the signaling transduction cascades controlled by G‐protein coupled receptors (GPCRs) are also involved in the regulation of resistance P450 gene expression (Li *et al*., 2013).

However, the expression and regulation of detoxification genes are less well reported in *Spodoptera exigua* (Hübner), which is a worldwide pest that damages numerous crops, including vegetables and ornamentals. Abamectin, lambda‐cyhalothrin, chlorantraniliprole, metaflumizone and indoxacarb have been extensively applied to control *S. exigua* in China, and very high resistance has been reported in field populations from Guangdong province in South China (Su & Sun, 2014; Tian *et al*., 2014). The enhanced breakdown of insecticides in resistance populations partially mediated these resistances, and increased P450 activity was the primary cause for metabolic resistance in this pest (Shimada *et al*., 2005; Tian *et al*., 2014; Wang *et al*., 2015, 2016). However, the molecular mechanism for multiple resistances of *S. exigua* to different insecticides remains to be illuminated, the genes for insecticide detoxification in this pest are also less well reported. There is still limited information about which genes respond to insecticide stress and resistance in *S. exigua*. To understand the response of *S. exigua* to insecticides, the CYP and UGT genes of this species were identified, the tissue‐specific expression patterns and the changes in expression levels of CYP and UGT genes under stress of abamectin, lambda cyhalothrin, chlorantraniliprole, metaflumizone or indoxacarb were analyzed.

## Materials and methods

### Insect culture

The insecticide‐susceptible strain of *S. exigua* was used in this study. This strain was provided by Wuhan Kernel Biopesticide Company, Hubei, China, in May 2001 and has been maintained in the laboratory without exposure to any insecticide. All stages of the insect were maintained in an incubator at 27 ± 1°C under a photoperiod of 16 : 8 h (L : D) and 50%–70% relative humidity (RH). The larvae were reared with artificial diet and adults were fed 10% honey solution (Lai & Su 2011).

### Insecticides

Chlorantraniliprole (95% technical grade [TG]) was acquired from Dupont (Wilmington, DE, USA). Indoxacarb (95% TG), metaflumizone (95% TG), and lambda‐cyhalothrin (95% TG) were provided by Nanjing Keweibang Chemical Co., Ltd, Zhejiang Xinnong Chemical Co., Ltd and Jiangsu Yangnong Chemical Co., Ltd, respectively. Abamectin (97% TG) was a gift from Hebei Weiyuan Bioengineering Co., Ltd.

### RNA extraction and cDNA preparation

For gene cloning, 3rd instar larvae were harvested. For gene tissue expression studies, head, fat body, midgut and Malpighian tubules were dissected in phosphate‐buffered saline from the 1‐day‐old 5th instar larvae using ribonuclease (RNase)‐free entomological scissors and tweezers (cleaned with RNaseZAP, Thermo Fisher Scientific, Wilmington, MA, USA). For insecticide induction studies of CYP450 and UGT genes, the *S. exigua* fat body cells were collected after treatment with insecticides. Total RNA from insects, tissues or cells was extracted using TRIZOL reagent (Invitrogen, Carlsbad, CA, USA) following the manufacturer's instructions. The quality and quantity of total RNA were determined by agarose gel electrophoresis and NanoDrop 1000 Spectrophotometer (Thermo Fisher Scientific), respectively. First‐strand complementary DNA (cDNA) was synthesized using Supermo III RT Kit (BioTeke, Beijing, China) with 3rd instar larvae RNA as the template for gene cloning. For 5′‐RACE (rapid amplification of cDNA ends) and 3′‐RACE, the SMART RACE cDNA Amplification Kit (Clontech, Mountain View, CA, USA) was used to generate both 5′‐RACE and 3′‐RACE amplification of cDNA from 3rd instar larvae RNA following the procedures described by the manufacturer.

### Cloning and nomenclature of the P450 and UGT genes

To identify putative P450 and UGT genes from the *S. exigua* transcriptome data (Pascual *et al*., 2012; Li *et al*., 2013), the P450 sequences of *D. melanogaster*, *Culex quinquefasciatus*, *Aedes aegypti*, *T. castaneum*, *Apis mellifera*, *B. mori*, *Plutella xylostella* and *S. litura* were retrieved from Cytochrome P450 Homepage (http://drnelson.uthsc.edu/CytochromeP450.html), GenBank (http://www.ncbi.nlm.nih.gov/) and Diamondback moth Genome Database (http://iae.fafu.edu.cn/DBM/index.php). The UGT sequences of *D. melanogaster*, *B. mori*, *Helicoverpa armigera* and *Zygaena filipendulae* were also retrieved from GenBank (http://www.ncbi.nlm.nih.gov/) and UDP Glucuronosyltransferase Homepage (http://www.flinders.edu.au/medicine/sites/clinical-pharmacology/ugt‐homepage.cfm). These insect P450 and UGT sequences were used for BLASTN (e‐value < 0.00001) searches of the *S. exigua* transcriptome data (Pascual *et al*., 2012; Li *et al*., 2013). Each hit was further screened using BLASTX (e‐value < 0.00001) against the non‐redundant database at the National Center for Biotechnology Information (NCBI) to confirm its identity with other insect P450 or UGT genes. The resultant sequences were considered as *S. exigua* P450 or UGT candidates with the screening protocol described by Han *et al*. (2016). The primers for fragment polymerase chain reactions (PCRs) (Table S3 and S4) were designed based on sequence information from *S. exigua* P450 or UGT candidates. PCR amplifications were carried out in the following conditions: initial denaturation at 94°C for 3 min, 35 cycles of 94°C for 30 s, 55°C for 30 s, and 72°C for 2 min, followed by the last elongation at 72°C for 10 min. The full‐length sequence of these genes was determined by 5′‐RACE and 3′‐RACE using SMART RACE cDNA Amplification Kit (Clontech, USA). The gene‐specific primers (Table S3 and S4) were designed based on sequences obtained from the internal fragments. All PCR products were purified using gel extraction kit (Omega, Doraville, GA, USA) and incorporated into the PMD‐19T vector (Takara, Kyoto, Japan) according to the manufacturer's protocol for sequencing. Professor David R. Nelson (member of the international Cytochrome Nomenclature Committee) and Professor Michael H. Court (member of UGT Nomenclature Committee) were requested to name all the P450s or UGTs from *S. exigua*, respectively.

### Multiple sequence alignments and conserved motifs analysis

Multiple sequence alignments for P450 and UGT sequences were made separately. Sixty‐seven P450 sequences and 32 UGT sequences of *S. exigua* were included for alignment. The alignments were made by ClustalX 2.0.12 using the default parameters, and the conserved motifs were displayed using WebLogo (http://weblogo.threeplusone.com/create.cgi). There are five conserved motifs in insect P450s, the helix C motif (WxxxR), the helix I motif (Gx[ED]T[TS]), the helix K motif (ExLR), the PERF motif (PxxFxP[ED]RE) and the heme‐binding motif (PFxxGxRxCx[GA]). The UGT signature motif which has been identified in a range of organisms is located in the middle of the C‐terminal domain: [FVA]‐[LIVMF]‐[TS]‐[HQ]‐[SGAC]‐G‐x(2)‐[STG]‐x(2)‐[DE]‐x(6)‐P‐ [LIVMFA]‐[LIVMFA]‐x(2)‐P‐[LMVFIQ]‐x(2)‐[DE]‐Q.

### Protein structure prediction

Twenty representative protein sequences from the *S. exigua* UGTs were aligned by Clustal X 2.0.12. Signal peptides were also predicted by SignalP3.0 on the CBS Prediction Servers (http://www.cbs.dtu.dk/services/SignalP-3.0/) (Bendtsen *et al*., 2004).

### Phylogenetic analyses

The P450s of *S. exigua* (*Se*), *T. castaneum* (*Tc*), *D. melanogaster* (*Dm*) and *B. mori* (*Bm*) were used for the phylogenetic analysis. Deduced amino acid sequences from these insects were aligned using Clustal X 2.0.12 (Larkin *et al*., 2007). Positions that have a high percentage of gaps and missing data were manually trimmed. The phylogenic tree was estimated by the neighbor‐joining method using MEGA 5.10 (Saitou & Nei, 1987; Tamura *et al*., 2011). Bootstrap analysis was performed using 1000 replicates to evaluate the significance of the nodes. For the comparative study of the UGTs, a phylogenic tree was also constructed with the UGTs from *S. exigua* (Se), *B. mori* (Bm), *H. armigera* (Ha) and *Z. filipendulae* (Zf), following the methods mentioned earlier.

### Cell culture and insecticide challenge

The fat body cell line of *S. exigua* was a gift from Dr. Huan Zhang (Institute of Zoology, CAS, Beijing, China). The cells were cultured and maintained at 27°C in SFX‐Insect serum‐free insect cell culture medium (HyClone, Logan, UT, USA) containing 10% fetal bovine serum (Life, Gaithersburg, MD, USA) in 25 cm^2^ cell culture flasks (Corning, Corning, NY, USA). Cells were passaged every 2–3 days as they reached confluence. Before experiments, cells were seeded in six‐well plates (TPP, Traisadingen, Switzerland) at 5 × 10^5^ cells/mL and left at 27°C for adhesion. For insecticide challenges, attached cells were treated for 12 h with insecticides (abamectin, lambda‐cyhalothrin, chlorantraniliprole, metaflumizone and indoxacarb), and then the cells were harvested for RNA isolation. The concentrations of insecticides for the challenge were determined with methylthiotetrazole (MTT) cell proliferation and cytotoxicity assay kit (Jiancheng Bioengineering Institute, Nanjing, China). Briefly, fat body cells (2 × 10^5^ cells/mL) were seeded in 96‐well culture plates and treated in triplicates with increasing concentrations for each of the insecticides for 12 h, and then the cells were loaded with MTT and incubated at 27°C for 4 h. The viability of fat body cells was determined according to the manufacturer's instructions. The maximum concentration without detected cytotoxicity for five insecticides was chosen for the challenge. Each of the five insecticides tested at 25 *μ*mol/L did not obviously affect the viability of fat body cells. Preliminary experiments also showed that fat body cells exposed to the 25 *μ*mol/L insecticide for 12 h led to significant increases in enzyme activities of their P450 and UGT activities (not shown). Three biological replicates per treatment were made, and the control incubations contained basal culture medium and the corresponding volume of dimethyl sulfoxide.

### Quantitative real‐time PCR analysis

The RNAs from larvae tissues or fat body cells were reverse transcribed using the HiScript^TM^ Q Select RT SuperMix (Vazyme, Nanjing, China). Purified RNA was subjected to DNase I to remove any residual genomic DNA according to the manufacturer's instructions. The mRNA abundance of P450 and UGT genes was estimated by quantitative real‐time PCR in an ABI 7500 Fast Real‐Time PCR System (Applied Biosystems, Foster City, CA, USA) using AceQ qPCR SYBR Green Master Mix kit (Vazyme, China) according to the manufacturer's protocol. Glyceraldehyde‐3‐phosphate dehydrogenase (GAPDH) was used as internal reference gene for analyses (Zhu *et al*., 2014). The PCR primers for P450 and UGT genes were designed using the Primer premier 5 software (Table S5 and S6). The specificity and sensitivity of the quantitative real‐time PCR assays were evaluated through melting curve analysis coupled with agarose gel electrophoresis (distinct single peaks in melting curve analysis, unique clear PCR product in agarose gel electrophoresis). PCR efficiencies were calculated from the standard curves. A reverse transcription negative control (without reverse transcriptase) and a non‐template negative control were included for each primer set to confirm the absence of genomic DNA and to check for primer‐dimer or contamination in the reactions, respectively. The PCR reaction system contained 0.8 *μ*L of the forward and reverse primers (10 mmol/L), 10 *μ*L 2 × SYBR Green Master Mix, 0.4 μL ROX Reference Dye 2, 1 *μ*L of the diluted cDNA samples and nuclease‐free water in a final volume of 20 *μ*L. The reaction conditions (a standard two‐step PCR amplification procedure) were as follows: an initial denaturation step of 95°C for 10 min, followed by 40 cycles of 95°C for 15 s and 60°C for 40 s. Melt curve analysis of the products was as follows: heating to 95°C for 15 s, decrease to 60°C for 60 s and then 95°C for 15 s. Three biological replicates were conducted for each sample. Relative expression levels of these genes were calculated by the 2^−∆∆^ ${}^{C_{T}}$ method (Livak & Schmittgen, 2001). All methods and data were confirmed to follow the MIQE (Minimum Information for publication of Quantitative real time PCR Experiments) guidelines (Bustin *et al*., 2009).

### Statistical analysis

The data from quantitative real‐time PCR were expressed as the mean ± standard deviation (SD) from triplicate biological replicates and were analyzed by using SPSS 16.0 software (SPSS Inc., Chicago, IL, USA). Student's *t*‐test followed by a two‐tailed paired *t*‐test was used to compare the significant differences between two samples. The statistical significance of the gene expressions was calculated using a one‐way analysis of variance followed by Tukey's multiple comparisons test for multiple sample comparisons; a value for change fold in expression level > two‐fold and *P* < 0.05 was considered statistically significant.

## Results

### Identification of the P450 and UGT genes in S. exigua

Queries for *S. exigua* P450 and UGT genes were done against the sequences from the other insects including *S. litura*, *H. armigera*, *Z. filipendulae*, *B. mori*, *P. xylostella*, *D. melanogaster*, *C. quinquefasciatus*, *A. aegypti*, *T. castaneum*, and *A. mellifera*. Sixty‐eight P450 and 32 UGT genes were found from the *S. exigua* transcriptome data (Table S1 and S2) and further validated by cloning using PCR, RACE‐PCR and sequencing.

**Figure 1:**
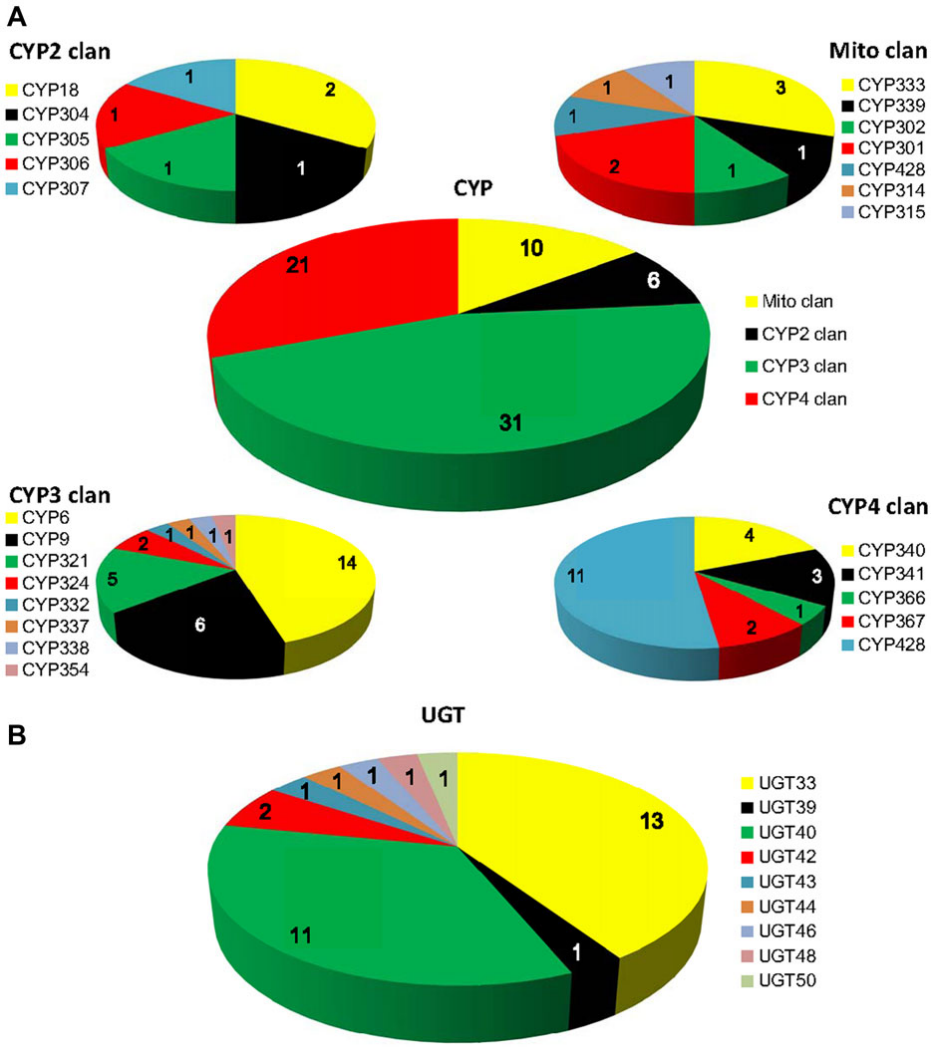
Pie chart showing the number and distribution of P450 (A) and UDP‐glucuronosyltransferase (UGT) genes (B) in *Spodoptera exigua*. (A) In the upper big pie chart, the red, green, yellow and black sections represent clans of CYP4, CYP3, Mito and CYP2, respectively. Four small pie charts describe the distribution of genes in the family. (B) In the pie chart, nine different colors represent nine families of the UGT, respectively.

According to the standard nomenclature, the 68 P450s were divided into 25 families and 40 subfamilies (Fig. 1A). The largest families included CYP4 with 11 genes and CYP6 with 14 genes. The average length of the P450 protein sequences with complete open reading frames (ORFs) was 514 amino acids. From the alignment analysis of P450 sequences, five highly conserved motifs were found in all the sequences: helix‐C (WXXXR), helix‐K (EXXR), helix‐I (Gx[ED]T[TS]), PERF (PxxFxP[ED]RE) and the heme‐binding (PFxxGxRxCx[GA]) (Fig. S1 A–E).

The 32 UGTs with complete ORFs were categorized into nine families (Fig. 1B). To maintain consistency with the nomenclatural rules, official names of the *S. exigua* UGTs reported here were approved by UGT Nomenclature Committee. The average length of the UGT protein sequence was about 521 amino acids. The largest families, UGT33 and UGT40 were composed of 13 genes and 11 genes, respectively. Multiple alignments of representative *S. exigua* UGT protein sequences revealed different patterns in the N‐ and C‐terminal domain: the N‐terminal substrate binding domain was more highly variable than the C‐terminal sugar‐donor binding domain (Fig. S2). The diversity of N‐terminal domain might be related to the detoxification or regulation of the various compounds. Several important domains distributed in the protein sequences were also predicted, including a signal peptide, signature motif, and a transmembrane domain. The signal peptide located at the N‐terminal end regulated the integration of the protein precursor into the ER compartment. The conserved protein sequence was found in the region of the UGT signature motif: [FVA]‐[LIVMF]‐[TS]‐[HQ]‐[SGAC]‐G‐x(2)‐[STG]‐x(2)‐[DE]‐x(6)‐P‐[LIVMFA]‐ [LIVMFA]‐x(2)‐P‐[LMVFIQ]‐x(2)‐[DE]‐Q (Fig. S1F). The transmembrane domain composed of about 16 hydrophobic amino acid residues, followed by a short cytoplasmic tail at the end of the C‐terminal domain.

### Phylogenetic analyses

**Figure 2:**
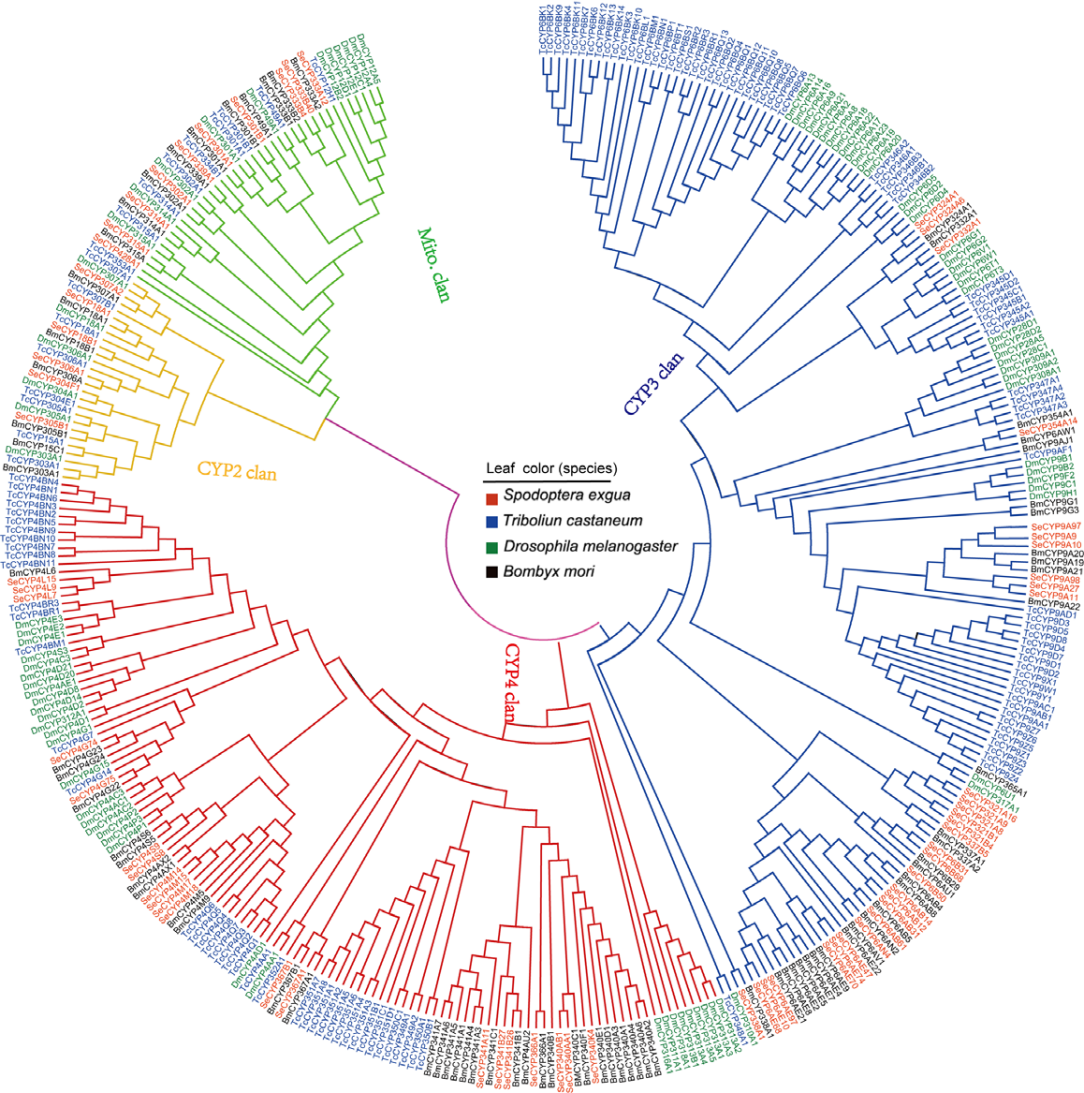
Phylogenetic tree of the P450s from four insect species. Deduced protein sequences of 84 *Bombyx mori* P450s, 85 *Drosophila melanogaster* P450s, 134 *Tribolium castaneum* P450s and 68 *Spodoptera exigua* P450s were aligned using Clustal X 2.0.12 and adjusted manually to minimize gaps. A consensus phylogenetic tree was constructed with MEGA 5.10, using the neighbor‐joining method. The four leaf colors represent the four species: red for *S. exigua*, blue for *T. castaneum*, green for *D. melanogaster* and black for *B. mori*. The tree is divided into four P450 clans: cytochrome P (CYP)2 (yellow), CYP3 (blue), CYP4 (red) and mitochondrial (green). Se, *S. exigua*; Tc, *T. castaneum*; Dm, *D. melanogaster*; Bm, *B. mori*.

A neighbor‐joining phylogenetic tree of *S. exigua* CYPs combined with CYPs from *B. mori* (84), *D. melanogaster* (85) and *T. castaneum* (134) was constructed to identify gene orthologs (Fig. 2). In general, the tree revealed four major clans in insects, including the CYP2, CYP3, CYP4 and mitochondrial clans.

The mitochondrial and CYP2 clans had relatively fewer gene expansions, and the size of the mitochondrial clan in insects was the same. In these two clans, there were a significant number of orthologous genes, which included CYP18A1, CYP306A1, CYP301A1, CYP302A1, CYP314A1 and CYP315A1. The branch showed that there was a high similarity between *B. mori* and *S. exigua*. CYP2 clan in insects was made up of a series of single‐member families such as CYP306, CYP304, CYP305, CYP303 and CYP15 families.

Genes in the CYP3 and CYP4 clans showed a variety of expansions in *S. exigua*. As a result, it was not easy to distribute orthologous relationships between these genes. Therefore, many species‐specific clusters were formed in the phylogenetic tree. In the CYP3 clan the two largest clusters were formed: the CYP6s and CYP9s, and in these two largest clusters small clusters were also formed, such as CYP6ABs, CYP6AEs, CYP6Bs and CYP9As. In the CYP4 clan the majority of CYPs from *S. exigua* were similar to that from *B. mori*; however, they were distant from *D. melanogaster* and *T. castaneum* in evolutionary relationships. Half of the genes in CYP4 clan from *S. exigua* were derived from the family CYP4, which were arranged into four subfamilies (CYP4M, CYP4L, CYP4S and CYP4G) with 2–4 members in each subfamily.

**Figure 3:**
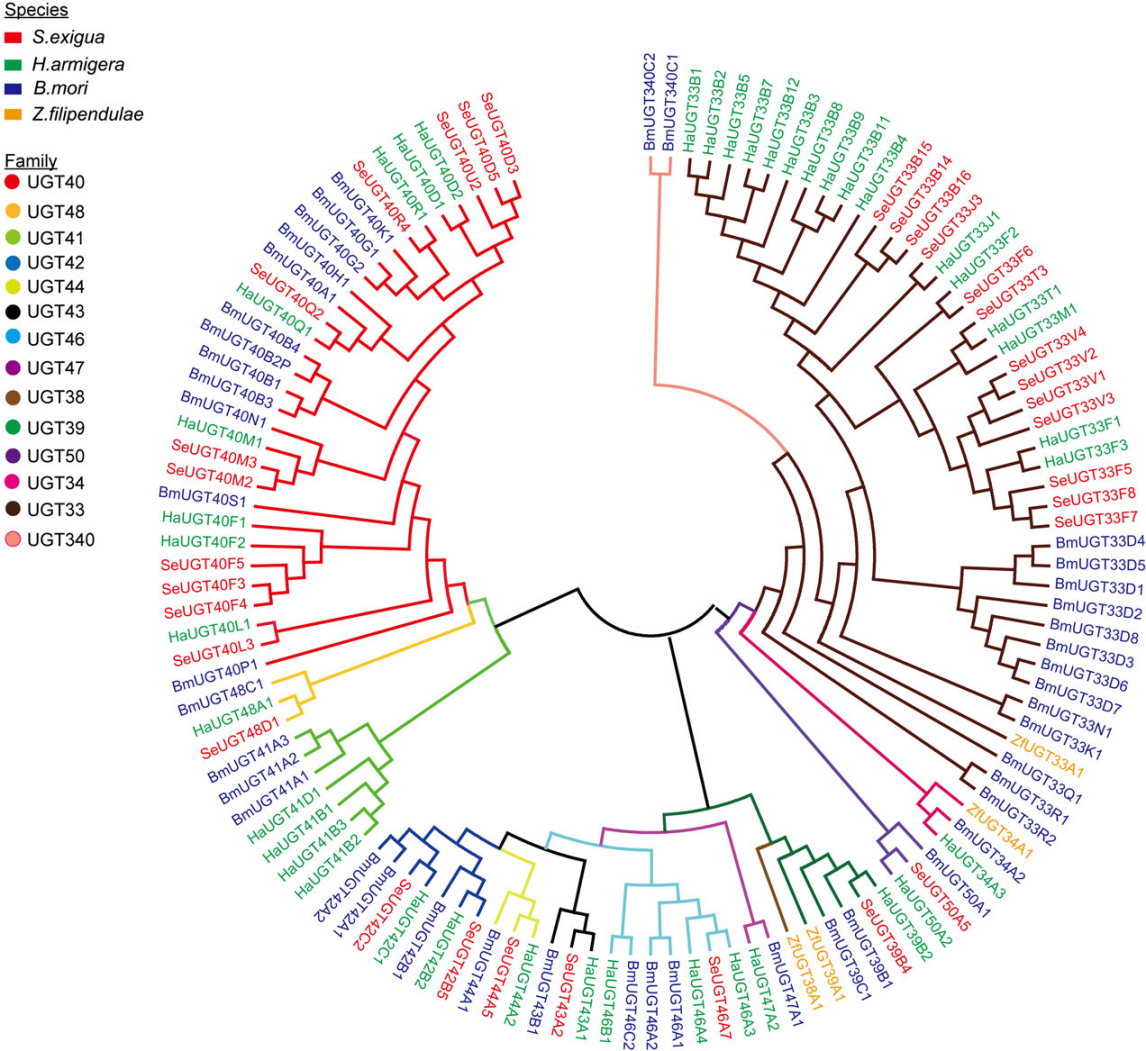
Phylogenetic relationship of the UDP‐glucuronosyltransferases (UGTs) of *Spodoptera exigua* and three other lepidopteran insect species. More than 120 UGT protein sequences from four lepidopteran insect species were aligned using Clustal X 2.0.12 and adjusted manually to minimize gaps. A consensus phylogenetic tree was constructed with MEGA 5.10, using the neighbor‐joining method. UGTs are highlighted by colored branches and names. Red, green, blue and yellow leaves stand for species of *S. exigua*, *Bombyx mori*, *Helicoverpa armigera* and *Zygaena filipendulae*, respectively. The nine different colors are used to distinguish the nine families of UGT. Se, *S. exigua*; Bm, *B. mori*; Ha, *H. armigera*; Zf, *Z. filipendulae*.

A phylogenetic tree using over 120 UGTs from *S. exigua* (32), *B. mori* (44), *H. armigera* (40) and *Z. filipendulae* (4), showed that there were variations in phylogenetic patterns in the UGT families (Fig. 3). UGT33, the largest family in Lepidoptera, was composed of 16 *H. armigera* UGTs, 13 *B. mori* UGTs, one *Z. filipendulae* UGT and 13 *S. exigua* UGTs. This family displayed a pattern of recent lineage‐specific gene divergence. The *S. exigua* UGTs from UGT33 family were diversified into five subfamilies. The second largest family UGT40 consisted of nine *H. armigera* UGTs, 12 *B. mori* UGTs and 11 *S. exigua* UGTs. There were seven subfamilies of *S. exigua* in this family. The branches showed that *S. exigua* and *H. armigera* were most similar in the evolutionary tree compared with other insect species. UGT43, UGT44, UGT48 and UGT50 from the lepidopteran species were single‐gene families, suggesting they have gone through evolutionarily independent paths without recent expansion. Conserved orthologous relationships among the lepidopterans were revealed in the above families. The UGT39 family also occurred in orthologous pairs; UGT39B2 (*H. armigera*) and UGT39B1 (*B. mori*) were orthologous genes of UGT39B4 (*S. exigua*).

### Tissue‐specific expression profiling

**Figure 4:**
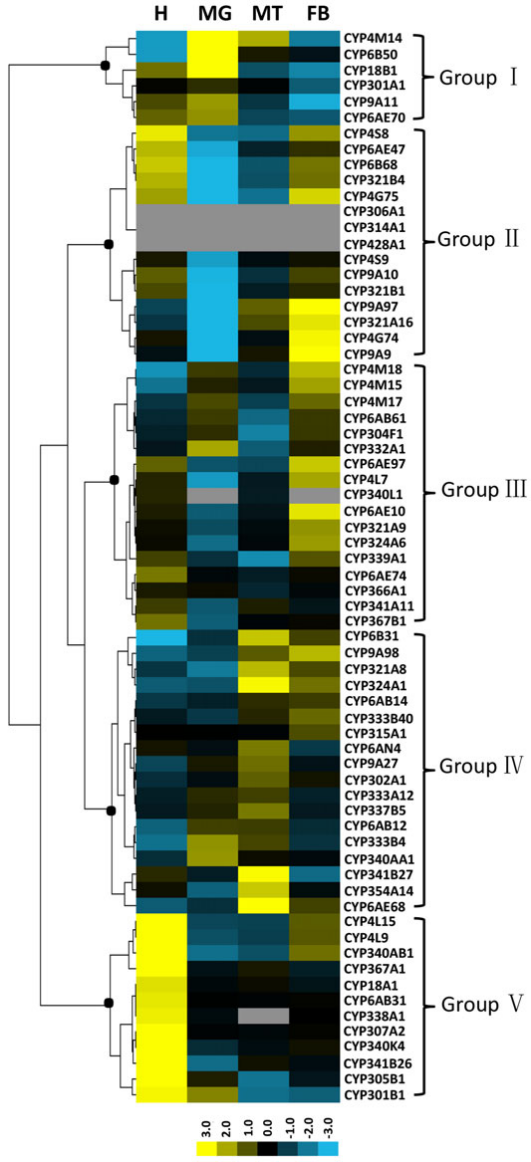
Tissue‐specific expression profiles of the P450 genes from *Spodoptera exigua*. Total RNA extracted from four major tissues and specific primers designed based on P450 sequences were used in quantitative real‐time polymerase chain reaction to quantify messenger RNA (mRNA) levels of all 68 P450s. Glyceraldehyde‐3‐phosphate dehydrogenase (GAPDH) was used as an internal reference gene. Relative mRNA levels were calculated by comparing the P450 mRNA levels to internal standard gene mRNA levels. Three independent replicates were carried out in each tissue. The mean normalized values were used to make the heat map using Cluster 3.0 and TreeView, and genes were clustered in terms of their expression patterns using the similarity metric of Euclidean distance and clustering method of complete linkage. In the map, yellow represents high expression values while blue represents low expression values. Gray denotes missing values. H, head; MG, midgut; MT, Malpighian tubule; FB, fat body.

The expression profiles of the *S. exigua* P450 and UGT genes identified in this study were determined by quantitative real‐time PCR. Specific primers were designed for each gene and the relative expression level of mRNAs was measured for each of them in the head, midgut, Malpighian tubules and fat body of the 1‐day‐old 5th instar larvae. The results showed that the P450 genes displayed diversified expression patterns in different tissues of larvae (Fig. 4). According to the expression patterns these genes were divided into five distinct groups by the hierarchical cluster analysis. Group I consisted of six genes (CYP4M14, CYP6B50, CYP18B1, CYP301A1, CYP9A11 and CYP6AE70) that showed higher expression levels in midgut than any other tissues. Nearly all of the genes in group II expressed higher expression levels in the head and fat body than in the other tissues. Group III comprised of 17 genes prominently expressed in the fat body. Group IV, with the most gene members, was highly expressed in the Malpighian tubules but had a relatively low expression in the head. Twelve genes from group V had higher expression levels in head compared to other tissues.

**Figure 5:**
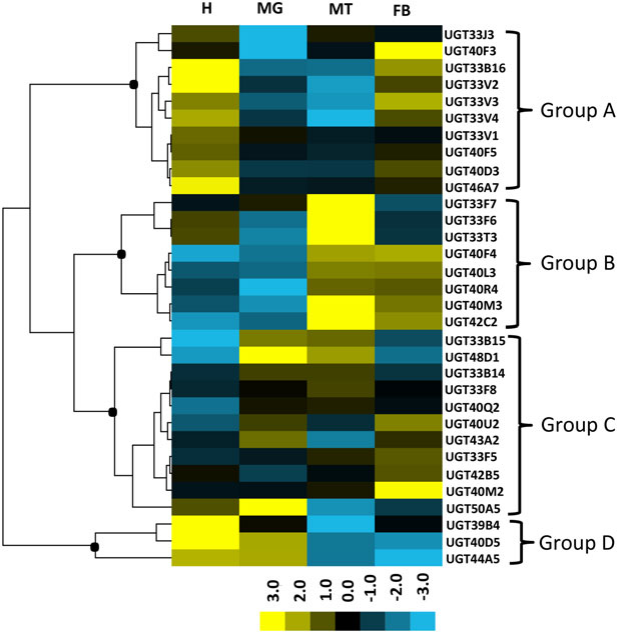
Tissue expression patterns of the UDP‐glucuronosyltransferases (UGTs) from *Spodoptera exigua* in different larval tissues. Total RNA isolated from four major tissues, and specific primers designed based on UGT sequences were used in quantitative real‐time polymerase chain reaction to quantify mRNA levels of all the 32 UGTs. Relative messenger RNA (mRNA) levels were calculated by comparing the P450 mRNA levels to internal standard glyceraldehyde‐3‐phosphate dehydrogenase (GAPDH) gene mRNA levels. For each tissue, there were three biological replicates. The mean normalized values were displayed using Cluster 3.0 program and TreeView, and genes were clustered in terms of their expression patterns using the similarity metric of Euclidean distance and clustering method of complete linkage. The yellow represents high expression values while blue represents low expression values. H, head; MG, midgut; MT, Malpighian tubule; FB, fat body.

Hierarchical clustering across different tissues was used to categorize UGT genes into four diverse groups (Fig. 5). Group A comprised of five UGT33 family genes, four UGT40 family genes and one UGT46 family gene with higher expression in head and fat body than the other two tissues. Group B comprised of eight genes from UGT33, UGT40, and UGT42 families, which had high expression values in Malpighian tubules, with UGT40 and UGT42 family genes also highly expressed in fat body. Group C containing most diverse genes from different families exhibited different expression patterns. Group D consisted of the remaining UGT39B4, UGT40D5 and UGT44A5 that expressed relatively high levels in the head, with the latter two also highly expressed in the midgut.

### Expression of CYP and UGT genes in response to insecticide challenges

**Figure 6:**
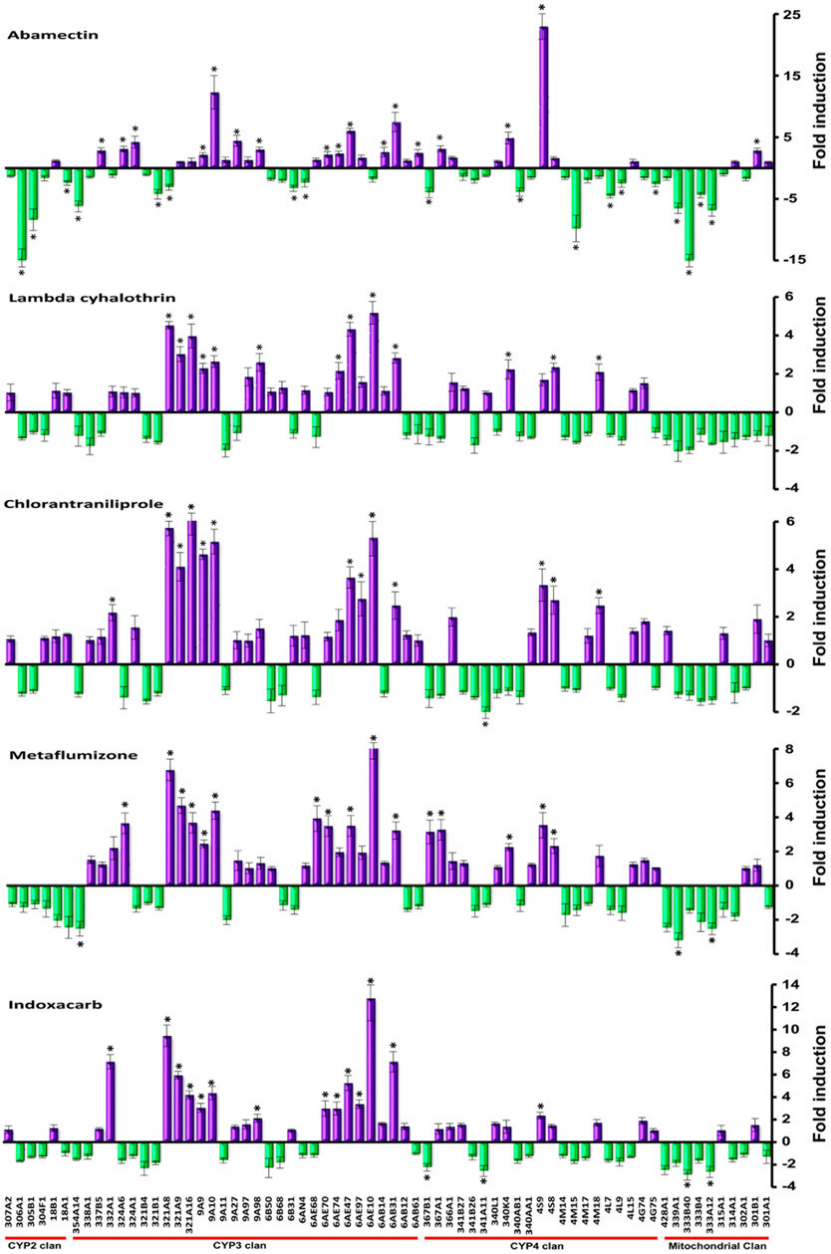
Effects of insecticide exposures on expression levels of P450 genes in fat body cells of *Spodoptera exigua*. The fold changes were acquired by comparing the P450 gene expression levels between treatment and the control. The expression level in control was considered the basal level, which was set to 1. The internal reference gene glyceraldehyde‐3‐phosphate dehydrogenase (GAPDH) for *S. exigua* was used to normalize expression levels. The values are expressed as means of two values. The error bars show the minimum and maximum values observed. Asterisks on the standard error bars indicate significant differences compared with the control. Positive values (purple column) represent the up‐regulated fold change of the cytochrome P (CYP)450 genes, and negative values (green column) represent the down‐regulated fold change of the CYP450 genes. A value of *P* < 0.05 and induction ratio > two‐fold were considered statistically significant.

**Figure 7:**
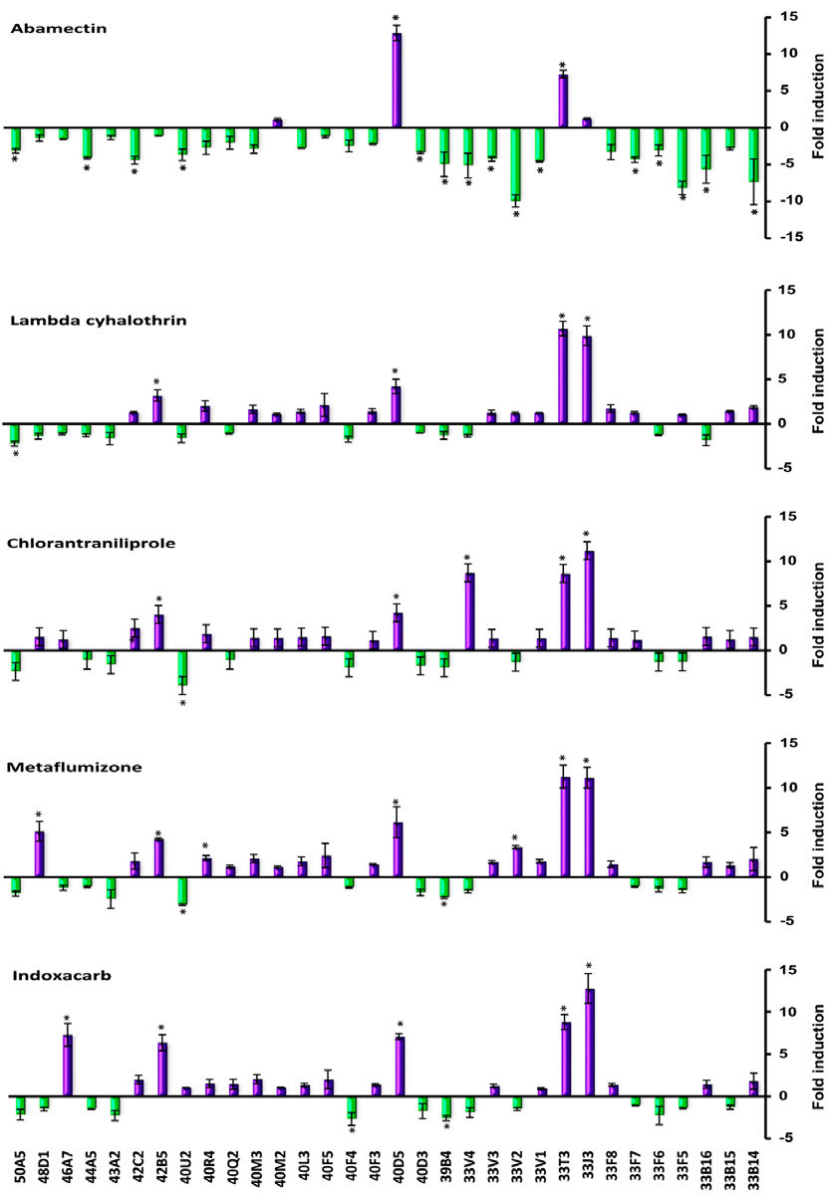
Effects of insecticide exposures on expression levels of UDP‐glucuronosyltransferase (UGT) genes in fat body cells of *Spodoptera exigua*. The fold changes were acquired by comparing the UGT gene expression levels between treatment and the control. The expression level in control was considered the basal level, which was set to 1. The internal reference gene glyceraldehyde‐3‐phosphate dehydrogenase (GAPDH) for *S. exigua* was used to normalize expression levels. The error bars show the minimum and maximum values observed. Asterisks above the standard error bars indicate significant differences compared with the control. Positive values (purple column) represent the up‐regulated fold change of the UGT genes, and negative values (green column) represent the down‐regulated fold change of the UGT genes. A value of *P* < 0.05 and induction ratio > two‐fold were considered statistically significant.

**Figure 8:**
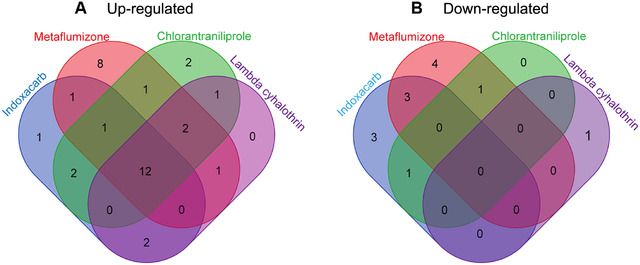
Venn diagram representing the overlapping of induction responses of *Spodoptera exigue* P450s and UDP‐glucuronosyltransferases (UGTs) by four insecticides. In the Venn diagram, the blue petal shows that the number of the P450s and UGTs were induced by indoxacarb. The other three colors are used to distinguish the remaining three insecticides: red for metaflumizone, green for chlorantraniliprole and purple for lambda‐cyhalothrin, respectively.

In order to understand the expression response of *S. exigua* to insecticides, quantitative real‐time PCR was performed to determine the change of transcriptional level for each of the 68 P450 and 32 UGT genes after the *S. exigua* fat body cells were exposed to abamectin, lambda‐cyhalothrin, chlorantraniliprole, metaflumizone and indoxacarb separately at 25 *μ*mol/L concentrations for 12 h (Fig. 6 and Fig. 7). The up‐ and down‐expression of CYPs and UGTs are summarized in Table 1. Five insecticide treatments significantly changed the expression level of CYPs and UGTs in fat body cells. The expression changes of CYP and UGT genes under each of the five insecticide stresses were significantly different (*F*_4,10_ = 86.295, *P* < 0.001 for CYPs and *F*_4,10_ = 550.225, *P* < 0.001 for UGTs), especially the transcriptional response of these genes under abamectin stress were significantly different from the other four small molecules (*α* = 0.05). More CYP and UGT genes were down‐regulated in expression level by abamectin than by the other four chemicals and no or only a few genes were significantly down‐regulated by lambda‐cyhalothrin, chlorantraniliprole, metaflumizone and indoxacarb. The change amplitude of expression level for CYP genes caused by abamectin was also greater than by others (Fig. 6). Meanwhile, similar induction responses for P450 and UGT gene expression were observed in fat body cells exposed to lambda‐cyhalothrin, chlorantraniliprole, metaflumizone or indoxacarb, which meant that the four smaller compounds exert similar stresses on fat body cells of *S. exigua*. The challenges by the four insecticides significantly increased the expression levels of CYP321A8, CYP321A9, CYP321A16, CYP9A9, CYP9A10, CYP6AE47, CYP6AE10, CYP6AB31, UGT42B5, UGT40D5, UGT33J3 and UGT33T3 (Fig. 6, Fig. 7 and Fig. 8). The up‐regulated P450 genes mainly belong to CYP3 clan and CYP4 clan. Most of the P450s in mitochondrial clan were down‐regulated under insecticide stresses.

**Table 1:** The expression changes of cytochrome P (CYP) and UDP‐glucuronosyltransferase (UGT) genes in fat body cells from *Spodoptera exigua* under insecticide stress

	Number and percentage of up or down expressions for CYPs (*n* = 68)			Number and percentage of up or down expression for UGTs (*n* = 32)		
Insecticide	Up	Down	Not significant	Up	Down	Not significant
Abamectin	17 (25%)	18 (26.5%)	33 (48.5%)	2 (6.2%)	15 (46.9%)	15 (46.9%)
Lambda‐cyhalothrin	13 (19.1%)	0 (0%)	55 (80.9%)	4 (12.5%)	1 (3.1%)	27 (84.4%)
Chlorantraniliprole	13 (19.1%)	1 (1.5%)	54 (79.4%)	5 (15.6%)	1 (3.1%)	26 (81.3%)
Metaflumizone	16 (23.5%)	3 (4.4%)	49 (72.1%)	7 (21.9%)	2 (6.3%)	23 (71.9%)
Indoxacarb	14 (20.6%)	4 (5.9%)	50 (73.5%)	5 (15.6%)	2 (6.3%)	25 (78.1%)

Abamectin treatment significantly increased the expression of 17 out of 68 CYP genes and two out of 32 UGT genes, and decreased the expression of 18 CYPs and 15 UGTs in fat body cells. The three greatest up‐expressed CYPs were CYP4S9 (23‐fold), CYP9A10 (12.3‐fold) and CYP6AB31 (7.5‐fold), and the three greatest down‐expressed CYPs were CYP333B40 (15‐fold), CYP306A1 (15‐fold) and CYP4M15 (9.8‐fold). Among the UGT genes, only two were up‐expressed (12.9‐fold for UGT40D5 and 7.3‐fold for UGT33T3). UGT33J3 and UGT40M2 were up‐expressed slightly compared with control. Most of the UGT genes were down‐expressed under abamectin stress and 46.9% of UGTs were significantly down‐regulated, of which the greatest down‐regulated gene was UGT33V2 (9.9‐fold).

Under lambda‐cyhalothrin stress, the expression levels of 32 CYP genes and 19 UGT genes were increased, among which 13 CYPs and four UGTs were significantly increased. Thirty‐six CYP and 12 UGT genes were slightly down‐regulated, and no CYP and only one UGT gene (UGT50A5, 2.2‐fold) had noticeable reduction in expression level. All the up‐expressed CYPs were derived from the CYP3 clan and CYP4 clan. All the mitochondrial clan members had slightly decreased expression after lambda‐ cyhalothrin exposure (Fig. 6).

The changes in expression levels of CYP and UGT genes under chlorantraniliprole stress were similar to that under lambda‐cyhalothrin stress. The up‐expressed CYPs and UGTs under lambda‐cyhalothrin stress were also increased in expression level under chlorantraniliprole stress. Four CYP genes including CYP321A16 (6.2‐fold), CYP321A8 (5.7‐fold), CYP6AE10 (5.3‐fold), CYP9A10 (5.2‐fold), and three UGT genes including UGT33J3 (11‐fold), UGT33V4 (8.7‐fold), UGT33T3 (8.6‐fold) were up‐expressed for over five‐fold (Fig. 6, Fig. 7). Compared with the control, only CYP341A11 (two‐fold) and UGT40U2 (3.9‐fold) were significantly down‐regulated.

Similar expression patterns of CYP and UGT genes in fat body cells when challenged with metaflumizone and chlorantraniliprole were also observed (Fig. 6 and Fig. 7). Sixteen CYPs and seven UGTs were up‐expressed and three CYPs and two UGTs were down‐expressed under metaflumizone stress. More than 70% of CYPs and UGTs were not significantly influenced at transcriptional level by this chemical (Table 1). The most up‐expressed CYPs and UGTs included CYP6AE10 (8.4‐fold), CYP321A8 (6.8‐fold), UGT33J3 (11.2‐fold), UGT33V4 (8.7‐fold) and UGT33T3 (8.6‐fold).

The changes in CYP and UGT expression levels induced by indoxacarb were similar with that by lambda‐cyhalothrin, chlorantraniliprole and metaflumizone. The up‐expressed CYPs belong to CYP clan 3 and clan 4. The CYPs and UGTs with over five‐fold increase in expression levels were CYP6AE10 (12.8‐fold), CYP321A8 (9.5‐fold), CYP6AB31 (7.2‐fold), CYP332A1 (7.2‐fold), CYP321A9 (6.0‐fold), CYP6AE47 (5.3‐fold), UGT33J3 (12.8‐fold), UGT33T3 (8.8‐fold), UGT46A7 (7.3‐fold), UGT40D5 (7.1‐fold) and UGT42B5 (6.3‐fold).

## Discussion

### Identification of CYP and UGT genes from S. exigua

P450 and UGT genes code for important detoxification enzymes, confer insecticide resistance in many resistance cases, and also respond to xenobiotic stress (Li *et al*., 2007; Liu *et al*., 2015; Krempl *et al*., 2016). However, the systemic analysis on families of these detoxification enzymes has been made only in a limited number of insect species. The gene families for detoxification in *S. exigua* were not intensively studied. In the present study, 68 P450 and 32 UGT genes expressed in larvae of *S. exigua* were identified. The P450s identified from *S. exigua* were classified into 25 families compared with *B. mori*, in which 84 P450s were categorized into 26 families (Ai *et al*., 2011). UGT families were also analyzed according to their protein sequences; the number (32) of UGT genes expressed in larvae of *S. exigua* was similar to that of *D. melanogaster* (34) (Luque & O'Reilly, 2002) and *A. aegypti* (34) (Ahn *et al*., 2012), but less than *B. mori* (45) (Huang *et al*., 2008) and *H. armigera* (42) (Ahn *et al*., 2012). Due to the lack of genomic data for *S. exigua*, the transcriptome data were very limited for this species, and only larvae were used to clone these genes in this report, the number of the P450 families and UGT families reported here might be incomplete.

### Most of the CYP and UGT genes of S. exigua are expressed in a tissue‐specific pattern in larvae

In this study, head, fat body, midgut and Malpighian tubules were tested for tissue‐specific expression as a result of detoxification function in these tissues (Fig. 4 and Fig. 5). P450s expressed in these tissues may be involved in xenobiotic detoxification (Giraudo *et al*., 2015). Midgut serves as the first line in the detoxification of xenobiotics (Scott, 2008); CYP450 and UGT genes which showed high expression in this tissue might be involved in the detoxification of xenobiotics. Malpighian tubules are thought to be the primary organs of excretion in insects; CYP450 and UGT genes that had higher expression levels in Malpighian tubules than in other tissues, may be responsible for the metabolism and detoxification of both endogenous solutes and xenobiotics, such as insecticides, and might also be involved in immunity (Dow & Davies, 2006). It has been identified that P450s specifically expressed in heads probably have specific functions (Yu *et al*., 2015). In *T. castaneum*, CYP6BQ9 predominantly expresses in brains, and causes the majority of deltamethrin resistance (Zhu *et al*., 2010). CYP4G15 is predominantly expressed in the brain of 3rd larval instar in *D. melanogaster* and is supposed to be involved in ecdysteroid biosynthesis (Maïbèche‐Coisne *et al*., 2000). Most of group III genes exhibited relatively high expression values in the fat body, which are suggested to be an important metabolism organ in insects. The fat body plays major roles in the life of insects. It is a dynamic tissue involved in multiple metabolic functions, including lipid and carbohydrate metabolism, protein synthesis, and amino acid and nitrogen metabolism (Arrese & Soulages, 2010). CYP450 and UGT genes which showed high expression in this organ might be involved in these functions. Tissue‐specific expression patterns of P450s and UGTs may reflect their roles in the metabolism of endogenous and exogenous substances.

### Insecticide exposure alters the expression levels of multiple CYP and UGT genes

It has been frequently reported that the expression levels of CYP genes in insects are influenced by insecticide or xenobiotic challenge (Feyereisen & Lawrence, 2012; Zhu *et al*., 2016). Each of the five insecticides tested in this study caused significant changes in transcription levels of multiple CYP or UGT genes in fat body cells of *S. exigua*. Thirteen to 17 P450 genes were up‐regulated significantly under the stress of abamectin, lambda‐cyhalothrin, chlorantraniliprole, metaflumizone or indoxacarb, and some of the CYP genes were down‐regulated by these insecticides (Fig. 6, Fig. 8 and Table 1).

UGTs catalyze the conjugation of the small lipophilic compounds with sugar, and play important roles in phase II metabolism of xeno‐ and endobiotic compounds in living organisms (Bock, 2016). Transcriptome analyses revealed that some UGTs were over‐expressed in resistant strains or induced under xenobiotic stress. The expression of UGT2B17 could be induced significantly in susceptible *P. xylostella* by chlorantraniliprole, and it was also over‐expressed in chlorantraniliprole resistant populations, and RNA interference of this UGT increased the toxicity of chlorantraniliprole on larvae of diamondback moth (Li *et al*., 2017). In our research, two to seven UGT genes were highly up‐expressed by the tested insecticides (Fig. 7, Fig. 8 and Table 1). The up‐regulation in transcription level of CYP or UGT genes may be applied to the insecticide stress, and probably is related to insecticide resistance.

Our research also demonstrated that the expression level of a detoxification gene may be influenced by chemicals with diverse structures. The significant up‐regulation induced by the five different insecticides were observed in five of 68 CYP genes (CYP9A10, 6AE74, 6AE47, 6AE31 and 4S9) and two of 32 UGT genes (UGT40D5 and 33T3). We speculate these genes share a mechanism of their expression regulation. However, the regulation mechanism needs further investigation.

### The induction expression of CYP and UGT genes by abamectin was different from that by lambda‐cyhalothrin, chlorantraniliprole, metaflumizone or indoxacarb

More interestingly, we found that lambda‐cyhalothrin, chlorantraniliprole, metaflumizone and indoxacarb induced similar expression responses for P450 and UGT genes, but abamectin triggered a unique response in gene expression distinct from the other four chemicals (Fig. 6 and Fig. 7). The comparisons on induced expression of genes by different xenobiotics had been reported in insects. Willoughby *et al*. (2006) reported that caffeine and phenobarbital highly up‐regulated multiple P450 and GST gene expressions; in contrast, no P450, GST or esterase gene expression was induced by spinosad, diazinon, nitenpyram, lufenuron or dicyclanil, and only DDT triggered the low‐level induction of one GST and one P450 in *D. melanogaster*. Poupardin *et al*. (2008) analyzed the induced expression of 12 detoxification genes in *A. aegypti* exposed to xenobiotics, including insecticides and found CYP6M6 and CYP6M11 were specially induced by fluoranthene and copper, respectively, and none of the 12 genes was induced by more than two compounds. Several members of CYP6B, CYP321A, and CYP9A subfamilies were induced by plant chemicals in *S. frugipersa*, and only a few genes from CYP9A subfamily responded to insecticides (Giraudo *et al*., 2015). In these studies, different xenobiotics exhibited distinct induction responses in one species and revealed nonuniform induction pattern for the tested genes by different xenobiotics. Our study presented that four insecticides induced similar detoxification gene expressions in fat body cells of *S. exigua*. A similar phenomenon had been reported in human hepatoma cells challenged by 24 pesticides; CYP3A4 was highly induced by pyrethroid insecticides and also moderately induced by other insecticides such as organophosphates, carbamates and herbicides, and CYP2A6 and CYP2B6 were induced by all the three pesticide types (Abass *et al*., 2012). Abamectin belongs to macrocyclic lactone insecticides (Clark *et al*., 1995) and the resistance mechanism had been reported to be associated with the over‐expression of CYPs (Riga *et al*., 2014; Gao *et al*., 2016). In fat body cell of *S. exigua*, 17 CYP genes and two UGT genes were up‐induced significantly by abamectin; these up‐regulated genes might be associated with the detoxification of abamectin. The unique pattern of gene expression induced by abamectin suggests that abamectin triggers a defense mechanism different from that of the other four insecticides in insects.

In fact, the regulation mechanisms of detoxification enzymes have been studied, and the activations of transcription factors were important mechanisms regulating the expression levels of detoxifying enzymes in insects. Transcription factors, CncC and Maf, regulate expression of CYP6BQ genes responsible for deltamethrin resistance in *Tribolium castaneum* (Kalsi & Palli, 2015). Similarly, in *Leptinotarsa decemlineata*, CncC regulates multiple cytochrome P450 genes conferring adaptation to potato plant allelochemicals and resistance to imidacloprid (Kalsi & Palli, 2017). The nuclear receptors HR96 and BR‐C were identified as positive and negative transcriptional regulators of phenobarbital induction of CYP6D1, respectively (Lin *et al*., 2011). The activation of CncC/Maf by phenobarbital induced the expression of CYP6A2 in *Drosophila* (Jyoti *et al*., 2011). Recent reports revealed the induced expression of CYP6DA2 by gossypol in *Aphis gossypii* was regulated by AhR (Peng *et al*., 2017) and CncC (Peng *et al*., 2016). Forkhead box A transcriptional factor modulates insect susceptibility to *Bacillus thuringiensis* Cry1Ac toxin by regulating the expression of toxin‐receptor ABC‐C2 and ABC‐C3 genes (Li *et al*., 2017). Meanwhile, Li *et al*. (2015) found that the GPCR/cyclic andenosine 3′,5′‐monophosphate/protein kinase A‐mediated regulatory pathway governs P450 gene expression and P450‐mediated permethrin‐resistance in *Culex* mosquitoes. The expression regulation of detoxification genes in insects involves multiple and complex mechanisms. The distinct induction of detoxification enzymes in *S. exigua* by abamectin and the other four insecticides, and a similar induction by four different insecticides may be mediated by different or the same transcription factors, respectively. However, the induction mechanism needs further investigation.

In summary, we found insecticide challenges alter the expression levels of multiple genes for detoxification; the response to abamectin in the expression of CYP and UGT genes is significantly different from the responses to other small molecular insecticides, and more CYP and UGT genes were down‐expressed by abamectin than by the other insecticides reported in this study. At the same time, a series of new issues arise from these results. What mechanisms underlie the distinct or similar induction responses to insecticides in insects? And does abamectin potentiate the toxicities of other insecticides via the suppression of detoxification genes at transcription levels?

## Disclosure

The authors have no conflict of interest.

## Supporting information


**Table S1** Cytochrome P450 genes of *Spodoptera exigua*.
**Table S2** UDP‐glucosyltransferase genes of *Spodoptera exigua*.
**Table S3** Primers used for amplification of Cytochrome P450 genes.
**Table S4** Primers used for amplification of UDP‐glucosyltransferase genes.
**Table S5** Primers used in quantitative real‐time PCR of Cytochrome P450 genes.
**Table S6** Primers used in quantitative real‐time PCR of UGT genes.
**Figure S1** The conserved motifs of the P450s and UGTs from *S. exigua*.
**Figure S2** Amino acid multiple alignments of the 20 representatives of *S. exigua* UGTs.
