# Supplementary material for: The expression of Spodoptera exigua P450 and UGT genes: tissue specificity and response to insecticides
Source: Insect Sci. 2017 Nov 21;26(2):199–216. doi: 10.1111/1744-7917.12538 (PMC7379962; doi:10.1111/1744-7917.12538)
Supplement: Supplementary file 1 — Table S1 Cytochrome P450 genes of Spodoptera exigua. Table S2 UDP‐glucosyltransferase genes of Spodoptera exigua. Table S3 Primers used for amplification of Cytochrome P450 genes. Table S4 Primers used for amplification of UDP‐glucosyltransferase genes. Table S5 Primers used in quantitative real‐time PCR of Cytochrome P450 genes. Table S6 Primers used in quantitative real‐time PCR of UGT genes. Figure S1 The conserved motifs of the P450s and UGTs from S. exigua. Figure S2 Amino acid multiple alignments of the 20 representatives of S. exigua UGTs. [file INS-26-199-s001.docx]

**Supplementary Information**

**The expression of *Spodoptera exigua* P450 and UGT genes: tissue specificity and response to insecticide**

Bo Hu, Shuheng Zhang, Miaomiao Ren, Xiangrui Tian, Qi Wei, David Kibe Mburu，Jianya Su*

Key Laboratory of Integrated Management of Crop Diseases and Pests (Ministry of Education), College of Plant Protection, Nanjing Agricultural University, Nanjing, 210095, China.

**This file includes:**

Table: S1-S6

Figure: S1-S2**Table S1 Cytochrome P450 genes of *Spodoptera exigua***

| **No.** | **Name** | **GenBank**  **Accession** | **Protein**  **length (aa)** | **No.** | **Name** | **GenBank**  **Accession** | **Protein**  **length (aa)** |
| --- | --- | --- | --- | --- | --- | --- | --- |
| 1 | CYP6AB12 | KX443422 | 513 | 35 | CYP305B1 | KX443454 | 487 |
| 2 | CYP6AB14 | KX443423 | 518 | 36 | CYP306A1 | KX443455 | 539 |
| 3 | CYP6AB31 | KX443424 | 512 | 37 | CYP307A2 | KX443456 | 539 |
| 4 | CYP6AB61 | KX443425 | 511 | 38 | CYP340AA1 | KX443457 | 490 |
| 5 | CYP6AN4 | KX443426 | 517 | 39 | CYP340AB1 | KX443458 | 507 |
| 6 | CYP6B31 | KX443427 | 504 | 40 | CYP340K4 | KX425014 | (501) |
| 7 | CYP6B50 | KX443428 | 504 | 41 | CYP340L1 | KX425015 | (200) |
| 8 | CYP6B68 | KX443429 | 503 | 42 | CYP4G74 | KX443459 | 563 |
| 9 | CYP6AE10 | KX443430 | 526 | 43 | CYP4G75 | KX443460 | 556 |
| 10 | CYP6AE47 | KX443431 | 532 | 44 | CYP4S8 | KX443461 | 493 |
| 11 | CYP6AE68 | KX443432 | 524 | 45 | CYP4S9 | KX443462 | 490 |
| 12 | CYP6AE70 | KX443433 | 541 | 46 | CYP4L7 | KX443463 | 493 |
| 13 | CYP6AE74 | KX443434 | 520 | 47 | CYP4L9 | KX443464 | 493 |
| 14 | CYP6AE97 | KX443435 | 525 | 48 | CYP4L15 | KX443465 | 492 |
| 15 | CYP9A97 | KX443436 | 531 | 49 | CYP4M14 | KX443466 | 503 |
| 16 | CYP9A9 | AB381883 | 536 | 50 | CYP4M15 | KX443467 | 502 |
| 17 | CYP9A10 | KX443437 | 531 | 51 | CYP4M17 | KX443468 | 501 |
| 18 | CYP9A11 | KX443438 | 530 | 52 | CYP4M18 | KX443469 | 499 |
| 19 | CYP9A27 | KX443439 | 528 | 53 | CYP341A11 | KX443470 | 510 |
| 20 | CYP9A98 | KX443440 | 531 | 54 | CYP341B26 | KX443471 | 508 |
| 21 | CYP321A8 | KX443441 | 495 | 55 | CYP341B27 | KX425016 | (495) |
| 22 | CYP321A9 | KX443442 | 497 | 56 | CYP366A1 | KX425017 | (411) |
| 23 | CYP321A16 | KX443443 | 497 | 57 | CYP367A1 | KX443472 | 496 |
| 24 | CYP321B1 | KX443444 | 495 | 58 | CYP367B1 | KX443473 | 500 |
| 25 | CYP321B4 | KX443445 | 511 | 59 | CYP301A1 | KX443474 | 529 |
| 26 | CYP324A1 | KX443446 | 505 | 60 | CYP301B1 | KX443475 | 537 |
| 27 | CYP324A6 | KX425013 | (494) | 61 | CYP302A1 | KX443476 | 507 |
| 28 | CYP332A1 | KX443447 | 503 | 62 | CYP314A1 | KX443477 | 517 |
| 29 | CYP337B5 | KX443448 | 492 | 63 | CYP315A1 | KX443478 | 482 |
| 30 | CYP338A1 | KX443449 | 465 | 64 | CYP333A12 | KX443479 | 511 |
| 31 | CYP354A14 | KX443450 | 513 | 65 | CYP333B4 | KX443480 | 530 |
| 32 | CYP18A1 | KX443451 | 538 | 66 | CYP333B40 | KX443481 | 504 |
| 33 | CYP18B1 | KX443452 | 518 | 67 | CYP339A1 | KX443482 | 578 |
| 34 | CYP304F1 | KX443453 | 511 | 68 | CYP428A1 | KX443483 | 540 |

Parentheses indicate partial sequences.

**Table S2 UDP-glucosyltransferase genes of *Spodoptera exigua***

| **No.** | **Name** | **GenBank**  **Accession** | **Protein**  **length(aa)** | **No.** | **Name** | **GenBank**  **Accession** | **Protein**  **length (aa)** |
| --- | --- | --- | --- | --- | --- | --- | --- |
| 1 | UGT33B14 | KU680280 | 518 | 17 | UGT40F3 | KU680296 | 522 |
| 2 | UGT33B15 | KU680281 | 516 | 18 | UGT40F4 | KU680297 | 524 |
| 3 | UGT33B16 | KU680282 | 515 | 19 | UGT40F5 | KU680298 | 522 |
| 4 | UGT33F5 | KU680283 | 524 | 20 | UGT40L3 | KU680299 | 520 |
| 5 | UGT33F6 | KU680284 | 521 | 21 | UGT40M2 | KU680300 | 521 |
| 6 | UGT33F7 | KU680285 | 524 | 22 | UGT40M3 | KU680301 | 520 |
| 7 | UGT33F8 | KU680286 | 519 | 23 | UGT40Q2 | KU680302 | 523 |
| 8 | UGT33J3 | KU680287 | 518 | 24 | UGT40R4 | KU680303 | 522 |
| 9 | UGT33T3 | KU680288 | 520 | 25 | UGT40U2 | KU680304 | 521 |
| 10 | UGT33V1 | KU680289 | 523 | 26 | UGT42B5 | KU680305 | 514 |
| 11 | UGT33V2 | KU680290 | 522 | 27 | UGT42C2 | KU680306 | 508 |
| 12 | UGT33V3 | KU680291 | 522 | 28 | UGT43A2 | KU680307 | 516 |
| 13 | UGT33V4 | KU680292 | 520 | 29 | UGT44A5 | KU680308 | 526 |
| 14 | UGT39B4 | KU680293 | 521 | 30 | UGT46A7 | KU680309 | 526 |
| 15 | UGT40D3 | KU680294 | 523 | 31 | UGT48D1 | KU680310 | 532 |
| 16 | UGT40D5 | KU680295 | 529 | 32 | UGT50A5 | KU680311 | 544 |

**Table S3 Primers used for amplification of Cytochrome P450 genes.**

| **Gene** | **primer name** | **Sequences(5’-3’)** | **primer name** | **Sequences(5’-3’)** |
| --- | --- | --- | --- | --- |
| CYP4G75 | CYP4G75F | GTGAGACACACTCAGTGTCGTTTG | CYP4G75R | CTTATTCGTACAACGTAAAAAGGGTA |
| CYP4G74 | CYP4G74F | TAATTGTGATTGGACCACGGGTTG | CYP4G74R | GTAAACGCGCAATAACACCATTGAC |
| CYP4L15 | CYP4L15F | ATGATTTTGTTATTAGTATCTGTTGT | CYP4L15R | TGTACACAGTACTACATTGACTCA |
| CYP4L7 | CYP4L7F | CTTTGCCTATCATCGGCAATGCT | CYP4L7R | GCATATATCAACATTTACACATT |
|  | 5'-CYP4L7GSP | CCTGTAGCTGTGAGTAGACCCTT | 5'-CYP4L7NGSP | CCGTACTTATCTGCGTAATGCGC |
| CYP4L9 | CYP4L9F | CTTTGCCTATCATCGGCAATGCTC | CYP4L9R | TGGTGATCTTGAGTTCCATCATCG |
|  | 5'-CYP4L9GSP | TGCAGAATGTTGAAGTGGAATGC | 5'-CYP4L9NGSP | ACGTCATCAGGCTATATGGACCT |
|  | 3'-CYP4L9GSP | TCACAAGGGACTGTGAGGTTGGA | 3'-CYP4L9NGSP | ATTCAACGTCAACCAGACATGTT |
| CYP4M18 | CYP4M18F | TGGACGTTTTAAAATGTTCTGGT | CYP4M18R | TGAAAAACCTTACATTACTGTCG |
| CYP4M17 | CYP4M17F | TGMACGAGCYMGRMTGATNAAGAAG | CYP4M17R | CATYTCCARCATGGCRAACTTTTG |
|  | 5'-CYP4M17GSP | AATGACTACAGCAGAGAACGGAT | 5'-CYP4M17NGSP | TCATCAGCTCAACTGGATCGCAT |
|  | 3'-CYP4M17GSP | TATTAAGGAGTCGCTGCGAATGT | 3'-CYP4M17NGSP | ACGACTTGCATCATCGAGAGGAT |
| CYP4M15 | CYP4M15F | TCGAMTGATCAGAAAAATACCCGG | CYP4M15R | CTCGTTTGAYAAAATTGACGAGCAC |
|  | 5'-CYP4M15GSP | TCGCCGTACTTCATTCCAGACAT | 5'-CYP4M15NGSP | ACGAATGACAGTCCATCCATTGC |
|  | 3'-CYP4M15GSP | GCGATGATGGAGATGAAGATAGC | 3'-CYP4M15NGSP | ACCTTCACTGCTGACTTAGTACT |
| CYP4M14 | CYP4M14F | ATACGTTCTCCAGTTGAAACCATGC | CYP4M14R | GTAATGTCGGAGGGACGCGTGACTG |
|  | 5'-CYP4M14GSP | ATTTTGCACCGTTACTAACGAGT | 5'-CYP4M14NGSP | TATCTCAATATCCTCAGGATTGT |
|  | 3'-CYP4M14GSP | CGGAGAATAGCGTCGGTAGACAT | 3'-CYP4M14NGSP | ATGATAGAGATGAAGATAGCAGT |
| CYP4S8 | CYP4S8F | TGRTATGGYTGCTATTCTTCAAAGA | CYP4S8R | AAMGGCACRTAMGCGTAGGGATGC |
|  | 5'-CYP4S8GSP | AACACCTTGAACACATAGCGGTC | 5'-CYP4S8NGSP | GCTCCCAAGCGTTCCCTATCCAT |
|  | 3'-CYP4S8GSP | TAAGTTGGGCGATCTACTAGTGA | 3'-CYP4S8NGSP | TCTACGAGCTGCACCGTCGAGCT |
| CYP4S9 | CYP4S9F | GCTGTTTGTGATAGTGTTTGGTGG | CYP4S9R | ATTCTTATTTATCTAAGATGCATT |
| CYP6AB61 | CYP6AB61F | CTTTACTTCTACGGAACGCGGAC | CYP6AB61R | TTCTCRTCTCTRTGNATAGCTTG |
|  | 5'-CYP6AB61GSP | TCACTAATTCTGGATCAGTACCT | 5'-CYP6AB61NGSP | TCAGTAGCCATCATACACATACT |
|  | 3'-CYP6AB61GSP | CCTACGATGCTATCAAAGAGATG | 3'-CYP6AB61NGSP | TACTTGATTCGACAGTGCTGTGT |
| CYP6AB12 | CYP6AB12F | CAACATGATTWTCATAGCTATAGC | CYP6AB12R | AAACACTTGAGCAGCAATTAATGC |
|  | 5'-CYP6AB12GSP | TCGTCAGCTACCTGCGTCATACT | 5'-CYP6AB12NGSP | ATGGCTTGTCATGCTTGACTCCT |
|  | 3'-CYP6AB12GSP | TCTAATGCTGGAGTGTAAACAGA | 3'-CYP6AB12NGSP | GATGCATTAATTGCTGCTCAAGT |
| CYP6AB31 | CYP6AB31F | AGGAACTTCAAGTACTGGGAGGA | CYP6AB31R | AAGCGYTCACCTATRCAAGCTCT |
|  | 5'-CYP6AB31GSP | TCATTCTCTGTCTGAGCAGCTTC | 5'-CYP6AB31NGSP | ACCTCGTCAGCTACTTGTGTCAT |
|  | 3'-CYP6AB31GSP | CAGAGAGATGCACTACTTGGAGA | 3'-CYP6AB31NGSP | GGGTTCCTGTTGCGACAGTGTAC |
| CYP6AB14 | CYP6AB14F | AAGTCGCGCGTCGAACGCACCATG | CYP6AB14R | TTGTCCATCACAGCCACTGCTCGG |
| CYP6AE10 | CYP6AE10F | AAGCTTCCAAACATGATCACGCT | CYP6AE10R | CTCTGAGTACGACGGTAAGACAT |
| CYP6AE97 | CYP6AE97F | ATGAACGGCCTTATCGTTCTCGCG | CYP6AE97R | GCCAATTCCACTTTATATTTCTTC |
|  | 3'-CYP6AE97GSP | GGCTTAAAGCTGGACGTAGGTGT | 3'-CYP6AE97NGSP | TTGGATCTGGACCTAGACTATGT |
| CYP6AE47 | CYP6AE47F | ACMTTCTTAYTGGGAAATTATGG | CYP6AE47R | TCTGGACGRAACACTTCAGGTTC |
|  | 5'-CYP6AE47GSP | CTGACGGAGTACTCTCCAGTTGT | 5'-CYP6AE47NGSP | TTGGGTATAAGTACAGGTTCTGT |
|  | 3'-CYP6AE47GSP | TCGATGCGTATTTAGCTCGTCAT | 3'-CYP6AE47NGSP | TGGAAGACTACATAATGCCTGAT |
| CYP6AE74 | CYP6AE74F | GTCCTRATTCCTAAAGATCCAGA | CYP6AE74R | CCAAACGGYAAATATACATACGG |
|  | 5'-CYP6AE74GSP | CTCTATCTATCATAGCTTCATAT | 5'-CYP6AE74NGSP | ATCACCATATGTGCCGAAGAGGT |
|  | 3'-CYP6AE74GSP | GGACGCTGTCATAGACGAGACAT | 3'-CYP6AE74NGSP | TGGAGGACTATACTATGCCCGAT |
| CYP6AE70 | CYP6AE70F | TAGTCAAACGCTAGCAGTACAGT | CYP6AE70R | GTAAACAAGTTCATGATAGGCT |
|  | 5'-CYP6AE70GSP | CTTCCTTGTGATTGTGCTCTGAT | 5'-CYP6AE70NGSP | TCCGTACCGAAGTACGCTCCAAC |
| CYP6AE68 | CYP6AE68F | ATGATTGCGCTGTATTTGTTGCTA | CYP6AE68R | AAACGCACTCTCGACACAGCAGAA |
| CYP9A9 | CYP9A9F | TACGCGGGCATTTCGTCCAGACAG | CYP9A9R | TGGTACTAATGTTAGCCAGCACAA |
| CYP6AN4 | CYP6AN4F | CACCAGRAACCAYGACTAYTGGGAG | CYP6AN4R | GCACMRTGTTACTKCCRTGGTTGA |
|  | 5'-CYP6AN4GSP | TCCTCCTGACTACATCCAGGTCT | 5'-CYP6AN4NGSP | TATTGTACAACTCTACAGATACT |
|  | 3'-CYP6AN4GSP | ACGATACGTCTGCTGACAGACAT | 3'-CYP6AN4NGSP | TTAGCAGGATTAGCAGCATTACT |
| CYP6B31 | CYP6B31F | GTCATAGCACATTATGCGTTAAAT | CYP6B31R | GTAGGTATTTATTTCTATAACATAG |
| CYP6B68 | CYP6B68F | TTCAACTATTGGAAGAGTAGAAAT | CYP6B68R | GAATGTGCTCGATATAGAACTATG |
|  | 5'-CYP6B68GSP | CTGAATTCCACCCCACGATCACT | 5'-CYP6B68NGSP | TCATCCTGTAGATCCCGACCACT |
| CYP6B50 | CYP6B50F | ATTAGCGACTACGGTGACATCTAC | CYP6B50R | ACTAATATGACTTATCTCTACTAA |
| CYP9A97 | CYP9A97 | GTGAAAAAGATCACTGTCAAAGAC | CYP9A97 | GGTCCAATTCCGAAGGGCATGTAC |
|  | 5'-CYP9A97GSP | ACCATTAGGCGCATCTTGGAGCT | 5'-CYP9A97NGSP | GCTGTTGTTTAAGAGGGAGCGAT |
|  | 3'-CYP9A97GSP | TTCGACCCTGAGCGGTTCTCAGA | 3'-CYP9A97NGSP | GCACACTCTTCATCCATTTACGT |
| CYP9A10 | CYP9A10F | GTCAAAGACTTCGAGTACTTCCTC | CYP9A10R | GGTCCAACTCCGAAGGGCATGTAC |
|  | 5'-CYP9A10GSP | GCAGTCTATGTCTATGGAGCCAT | 5'-CYP9A10NGSP | ATGAACGGTACCATCAGACGCAT |
|  | 3'-CYP9A10GSP | GGTCTATATGGATATGGTTGTGT | 3'-CYP9A10NGSP | TGACAGACTATGCAATAAGGACT |
| CYP9A27 | CYP9A27F | ACGGGGTGTTGTGTCTGAAGTTG | CYP9A27R | TCGCCATGAGACGCCATATGTAC |
| CYP9A98 | CYP9A98F | GATAAATCAACTGATGACGGAGCT | CYP9A98R | GGGTGGCTGGCAGTGGTTGGATGA |
|  | 5'-CYP9A98GSP | CAGTAAGAACGACATACTCGATG | 5'-CYP9A98NGSP | AATGCTTGAGCCACCAAATCATC |
| CYP9A8 | CYP9A8F | ATTTGATACCTGAAGTTTGAG | CYP9A8R | TCATCAGGAGACATTTGGAAG |
|  | 5'-CYP9A8GSP | AGGAGAAACTACCATTTCTCGGA | 5'-CYP9A8NGSP | TGGTATGCAATTACTTTTATCTC |
| CYP9A11 | CYP9A11F | ATGATTATCTTTTTCATTTGGTTG | CYP9A11R | TTATTTTCTTTGTCTAAATCTAAG |
| CYP18A1 | CYP18A1F | GGTCTCGGCATCATCAATAGCGA | CYP18A1R | ATCTAACGTTCGGTCTACTATGT |
|  | 5'-CYP18A1GSP | CATCGTTATGCCGCAGATGACGT | 5'-CYP18A1NGSP | GTAAGTCATGCCGAATTCACGCA |
| CYP18B1 | CYP18B1F | CTTGGTGTCAAGGTGTGGCCGAAT | CYP18B1R | GATGTCAGGGACGTCTCCATTCCA |
|  | 5'-CYP18B1GSP | CATCATCAGCTGACATATGACAT | 5'-CYP18B1NGSP | AGCTCCACCATAAGCTCGTCAAT |
|  | 3'-CYP18B1GSP | ATCGTCCAGATCATGAATGATCT | 3'-CYP18B1NGSP | TTTCTGCTGGAATGGAGACGTC |
| CYP301A1 | CYP301A1F | CCGCCATGGGTCGTTCTCTCCG | CYP301A1R | ATATCATTRTCGAACTCRTGAGG |
|  | 3'-CYP301A1GSP | GTGAAGAAGTACGTGGCTCCCAT | 3'-CYP301A1NGSP | CCTCATGAGTTCGACAATGATAT |
| CYP302A1 | CYP302A1F | GGAGTACATTCCAGAAGAATTTCA | CYP302A1R | CATATATTTTGTTCTGCTAATCTC |
|  | 5'-CYP302A1GSP | ACGCTCGTTGAATGCTACAATAC | 5'-CYP302A1NGSP | TCTCTTATCACTATGTCAGTACT |
|  | 3'-CYP302A1GSP | GCAACTTACGTACGAGCTTGTAT | 3'-CYP302A1NGSP | CCTCTCGGATGCCACAGTATGTA |
| CYP304F1 | CYP304F1F | ACGGNGCNTTCTGGATAGTTCTGGC | CYP304F1R | CTTCTGCCTGCTCCAAANGGCAATGA |
|  | 5'-CYP304F1GSP | ACGGTTGACTCCAGTTCATCACT | 5'-CYP304F1NGSP | TGATGCCAGAAGTAACCGTCAGT |
|  | 3'-CYP304F1GSP | ACATACTCAGTGGACCAGCTGAT | 3'-CYP304F1NGSP | ATCCTCGTAGAGCGTCTGCTTCT |
| CYP305B1 | CYP305B1F | GGCTATAAGAATAAGGGTATAACT | CYP305B1R | TTTCATCGATGAATCTTTCAGGTT |
|  | 5'-CYP305B1GSP | ACTCGAATACATTACATCCAGGA | 5'-CYP305B1NGSP | TAACTTTTGAGATGTCTGCGTTC |
|  | 3'-CYP305B1GSP | CTGGTGTACACATCAGCGTTCAT | 3'-CYP305B1NGSP | AGGTACTACAGTACTTATGTCAT |
| CYP306A1 | CYP306A1F | GATTTATGGCATTGGTATGGGCAG | CYP306A1R | AAGACCAGCTCCAAACATATCAGC |
|  | 5'-CYP306A1GSP | CGTGACACGCTGTGCTTACTCAT | 5'-CYP306A1NGSP | GTTTCCATGCATGATACCGTGTG |
|  | 3'-CYP306A1GSP | TGAGTCATATGCTGACGAATTCT | 3'-CYP306A1NGSP | GTTCTAACACGTGGTCAAGCTCA |
| CYP307A2 | CYP307A2F | ATGAAYATGTTYTSCCACTACATG | CYP307A2R | ACTATTGTNCCYTTTTCNACTCC |
|  | 5'-CYP307A2GSP | TTGATCTCCCAGAAGATTTCGTC | 5'-CYP307A2NGSP | CTAACGTTGCACATGTAGTGGGA |
|  | 3'-CYP307A2GSP | GAATGTCTGAGATACGCTTCATC | 3'-CYP307A2NGSP | GGAGTAGAAAAAGGGACAATAGT |
| CYP314A1 | CYP314A1F | TGAGCTACCTATCGACACAGATA | CYP314A1R | CGCCATYTTTGCATTATCTTAGC |
|  | 3'-CYP314A1GSP | ATGGTAATTGCGGGACATAAGCT | 3'-CYP314A1NGSP | TTCTGGCGTGCTCGCGAGTACAT |
| CYP315A1 | CYP315A1F | GAYNNWCGTCAWAAACARTTAGG | CYP315A1R | GGCARWGAYGCWGAAGACACATG |
|  | 5'-CYP315A1GSP | CATGTTAACAGGCCATCCATACA | 5'-CYP315A1NGSP | ATAAGGCATCACTATGTGCGCTA |
|  | 3'-CYP315A1GSP | TGCATCTTCGGAGCTTACAAACT | 3'-CYP315A1NGSP | ATTAGGAAGAGATGAACAATACT |
| CYP321A16 | CYP321A16F | CTCACGACAAAAAATTAACGATGGT | CYP321A16R | CACAAAATTGTCTATTTCACATTTC |
| CYP321A9 | CYP321A9F | ACTGTTTGTYATYGACCCGAAAAATG | CYP321A9R | TGAYATTTCTNAGTCTTACATTAAG |
|  | 3'-CYP321A9GSP | AGACAGTGCGTTCAGAACACTGT | 3'-CYP321A9NGSP | GATAGGAGACCCAATGACGACAT |
| CYP321A8 | CYP321A8F | CAAAATGTTGTTTTTACCTTTGAG | CYP321A8R | TATGACACCGATTAGACATTTCT |
| CYP321B1 | CYP321B1F | TCAACGAGCTTAGACAAGTTCAGA | CYP321B1R | GCATCCATAGCTACATTACATACA |
| CYP321B4 | CYP321B4F | ATCAGTATTATTGGAAGAAACGTG | CYP321B4R | AAGCGTTCAGGATCAAATTTCTCA |
|  | 5'-CYP321B4GSP | ACCGTTGAGCATTAGGACATTGT | 5'-CYP321B4NGSP | TCAACTTGTGCGATGTACTGCAT |
|  | 3'-CYP321B4GSP | GCACACGAGGATGTAGATAGAGT | 3'-CYP321B4NGSP | ACGTATGATGCAGTTGGAGAAGT |
| CYP324A1 | CYP324A1F | CGTNGGCATSTGGNTRTTCTGGCG | CYP324A1R | ATRCAMWTCCTYGGTCCYTCYCC |
|  | 5'-CYP324A1GSP | GTGAACACCAACGTCAGACGTCT | 5'-CYP324A1NGSP | TCTGTTCCTGAACACATCAGCGT |
|  | 3'-CYP324A1GSP | AAGATTGACGATAAGCTGACTAT | 3'-CYP324A1NGSP | CAGCATGCAGATGGATCCTGAGT |
| CYP332A1 | CYP332A1F | GAGYCCAATGTGGRTNCARATGAGG | CYP332A1R | TGCATCATNCCGTAGCGTTTACCTA |
|  | 5'-CYP332A1GSP | GTATACGCTACCGTGTCTGATGT | 5'-CYP332A1NGSP | CCGTCGAGTTTATGTTCATAAGT |
|  | 3'-CYP332A1GSP | GTCTACGTGAACGTGGTATCCAT | 3'-CYP332A1NGSP | GAGTGGCGTCCTGAGAGATTCAT |
| CYP333A12 | CYP333A12F | TARARCRTTGAGTGANTGTTATAC | CYP333A12R | GAGTTTCAATCTCTAATTCRGCAA |
|  | 5'-CYP333A12GSP | CTGATCTATGTGTTCTGGCTCAT | 5'-CYP333A12NGSP | GTAGAGTAGACATCATGTCGAAT |
|  | 3'-CYP333A12GSP | ACATAAACGTTATCTGCGAGCGT | 3'-CYP333A12NGSP | GAGGAGGACAGATAGAGATCACT |
| CYP333B4 | CYP333B4F | GAAATTCCCGGACCTCCATCTTTGC | CYP333B4R | GCTATGCGACGACCWATRCAGCTTC |
|  | 5'-CYP333B4GSP | CCGTGATCAGTGACTAGACCAGT | 5'-CYP333B4NGSP | GCGTAATATATGTGCAGCAGCTT |
|  | 3'-CYP333B4GSP | CAGTGGTGTCAGGCAATCTTCGT | 3'-CYP333B4NGSP | AGTGTTCGCTCACAGAGACATGT |
| CYP333B40 | CYP333B40F | GTTGAGRCCGCTCTTCCAGTTG | CYP333B40R | GATCGTGGACGCATTGGATCAG |
|  | 5'-CYP333B40GSP | AGTTCCGCACTGTCAGGATCGCT | 5'-CYP333B40NGSP | CCATACTTATCGAAGAGTCTCAT |
|  | 3'-CYP333B40GSP | AAGAGTACAATGTGATGGGCTAC | 3'-CYP333B40NGSP | GCTCACAGAGACATGTCGCTGCT |
| CYP337B5 | CYP337B5F | GCAGTNTTRGCTGGTGAYTTCCAAAG | CYP337B5R | CATATTCTGTTTCCTTCWCCAAASGG |
|  | 5'-CYP337B5GSP | GATGGATATCGTCTTCAACCACT | 5'-CYP337B5NGSP | GACTTAGTTTATGCCTCAAGAGT |
|  | 3'-CYP337B5GSP | TGGACCAAGTGATTAATGAAGCT | 3'-CYP337B5NGSP | TGATATCACCATATGCCTTGCAT |
| CYP338A1 | CYP338A1F | CCAARTTCTTCAGCTACTGGTACGTC | CYP338A1R | CTTCRTCWTCCAARAATCTATCAGGA |
|  | 5'-CYP338A1GSP | GCCTTCGCGTAATAGGGTCCACT | 5'-CYP338A1NGSP | GTCCAAGGAGAGACCTCGACTAT |
|  | 3'-CYP338A1GSP | GTGCCTGAATGCATTGACTGCAT | 3'-CYP338A1NGSP | GATGAATATTTGGACGTGGCTAT |
| CYP340AA1 | CYP340AA1F | CTACAACTGTGTCCAAATGTTCT | CYP340AA1R | CAGTCATGATTTTTGCAGTGCTCA |
|  | 3'-CYP340AA1GSP | AAGCCATGTTCAGGGTGATTGAT | 3'-CYP340AA1NGSP | CTGTTAGATCAACTCCTAGAACT |
| CYP340AB1 | CYP340AB1F | ATWGGATCTYACCCAGAAGTAC | CYP340AB1R | TAGTTTCGTACGACATACGCAAT |
|  | 5'-CYP340AB1GSP | TTCTGGGTAAGATCCTATTGCTA | 5'-CYP340AB1NGSP | TGCTGTCTAATTTCTTCATCACT |
|  | 3'-CYP340AB1GSP | CGGTGTTAGTTACCACTCTATGT | 3'-CYP340AB1NGSP | GAGGTGGCTTGATCCTGCAAAGT |
| CYP340K4 | CYP340K4F | GTCATCAGGAGACATTTGGAAGCG | CYP340K4R | ATTTGATACCTGAAGTTTGAGATT |
|  | 5'-CYP340K4GSP | CAATGTGAACGCCGGACTCAGTA | 5'-CYP340K4NGSP | CTTCCAAATGTCTCCTGATGAC |
|  | 3'-CYP340K4GSP | AAGAACTAGTGGATACTTTACAT | 3'-CYP340K4NGSP | TCATTGAATCTCAAACTTCAGGT |
| CYP340L1 | CYP340L1F | TGAGATCAAAGAACATTTGAACAC | CYP340L1R | TTAGACGAGTGATGTCAGCAGTAA |
|  | 5'-CYP340L1GSP | AATCGTTCCTGYACATCTGGATA | 5'-CYP340L1NGSP | TGGTGTCATAAGCTGCTAATACA |
|  | 3'-CYP340L1GSP | CATGTGCTCTGTCGCTGTACGAT | 3'-CYP340L1NGSP | CGGACCGATGGCTGAATCCAGAT |
| CYP341A11 | CYP341A11F | TTYGCWCCAGTKTCMATCTGGCG | CYP341A11R | GACRAAGTTCTCTACTACTTTAGG |
|  | 3'-CYP341A11GSP | TAGAGAGAGTAGTCAAGGAGTCT | 3'-CYP341A11NGSP | TTCAGGCGTAGCGTGCTCAATAT |
| CYP341B26 | CYP341B26F | AGAAACCTTATCGGCAATGGAACT | CYP341B26R | ATATGCACCAGAACTCCACAACCA |
|  | 5'-CYP341B26GSP | GCAGTTTCACACACAGAATCCAT | 5'-CYP341B26NGSP | CTGTTCAGCCAGGACAGTGCTCT |
|  | 3'-CYP341B26GSP | TCTGGTGGAGAACGAGGGTATTC | 3'-CYP341B26NGSP | GGAGCATCATTCACCGTGTCCAT |
| CYP341B27 | CYP341B27 | CATACTCCTGCTGATACACCAGCG | CYP341B27 | TAGGTCCATAACTGAATGGCATGA |
|  | 5'-CYP341B27GSP | GACGCGGACGCCAAATAGGCACT | 5'-CYP341B27NGSP | CCGCTTCAGGATCAGCCACCATT |
|  | 3'-CYP341B27GSP | CTGAAGTACCTCGATGCTGTGAT | 3'-CYP341B27NGSP | GTACCTGGCACTGGTATCATGGT |
| CYP354A14 | CYP354A14F | CGTGGTATTCCATACAGAAAGCCA | CYP354A14R | TTAATGGTACAGAGATTTGGACCG |
|  | 5'-CYP354A14GSP | AAGTGTGCATTCTCATCGGACAT | 5'-CYP354A14NGSP | TTGTTTGCTTCATCTCTACATCT |
| CYP366A1 | CYP366A1F | ACAAACTCGTTTGTTGAGAAGCCA | CYP366A1R | TATTTCTTACCAATACAAGCTCGT |
|  | 5'-CYP366A1GSP | ATTGAACACGTCCTGGTAGCCAT | 5'-CYP366A1NGSP | GCAGTGACAAGTCCATCTCCGAG |
|  | 3'-CYP366A1GSP | ATGAGTTACTGTGAGGCAATCAT | 3'-CYP366A1NGSP | ATGAGGTACGCTGACAAGGATCT |
| CYP367B1 | CYP367B1F | TGTATCAAAATTCTGGCTCGGTCC | CYP367B1R | ATRACACTRAGYTSAGTATTAACAC |
|  | 5'-CYP367B1GSP | CTATCTTCCTATGTCGTCTCCAT | 5'-CYP367B1NGSP | CACCAAGGACATCAGCCATGTAT |
| CYP324A6 | CYP324A6F | ATCGTATCGCTATGGGTGTACTCA | CYP324A6R | AGGTACCTAAATGTATGTGAAGTA |
|  | 5'-CYP324A6GSP | CCTGTAGATCAGGTCTTCTACCT | 5'-CYP324A6NGSP | AGCGTCTACGGATCGATGTCCAT |
| CYP367A1 | CYP367A1F | CAACTGTGTATGATCACTTCAGTG | CYP367A1R | ACGATTATTCTTCCGTTTCTTGGT |
|  | 3'-CYP367A1GSP | CCTGAATCGTGACCCTCGCTACT | 3'-CYP367A1NGSP | ACGGCTCTCATCAAGACGCTAGT |
| CYP428A1 | CYP428A1F | GACAACACAGTCCGTAGTCAATAA | CYP428A1R | AGAATCCTTTCGAAAGACTTCGTC |
| CYP339A1 | CYP339A1F | TAGGTAGATTTCTTAGAGAAGAGA | CYP339A1R | TTAGTTGAAACCGCTTCTGTTCTG |
| CYP301B1 | CYP301B1F | GGTGCAGCAGCACAGTATACGGT | CYP301B1R | ATCGCTGTTCCCCATCACGTAGT |
|  | 5'-CYP301B1GSP | AAGGCATCTGCTACTTCAGCAAC | 5'-CYP301B1NGSP | ACCGTATACTGTGCTGCTGCACC |
|  | 3'-CYP301B1GSP | TCAAATGCCTTATCTGAAGGCGT | 3'-CYP301B1NGSP | TCGGTAATGGCCGACAGCTGACT |
|  | UPM(short) | CTAATACGACTCACTATAGGGC |  |  |
|  | UPM(long) | CTAATACGACTCACTATAGGGCAAGCAGTGGTATCAACGCAGAGT | | |
|  | NUP | AAGCAGTGGTATCAACGCAGAGT |  |  |

**Table S4 Primers used for amplification of UDP-glucosyltransferase genes.**

| **Gene** | **Primer Name** | **Sequences(5’-3’)** | **Primer Name** | **Sequences(5’-3’)** |
| --- | --- | --- | --- | --- |
| UGT33B14 | 33B14F | ATAACAGTTTGGAACACCGGCGTG | 33B14R | GCACGTGTTCCGTCCACCATAT |
|  | 3'-33B14GSP | TTAGCTGTAGTTCTAGCCATTAC | 3'-33B14NGSP | TTGACGGTCTTGTCGTGGTTAC |
| UGT33B15 | 33B15F | GTATTCTTGCGGCATCCATTCAAT | 33B15R | GGAATTCCTACTAGAGGTACTCC |
|  | 3'-33B15GSP | GTTCTCTCAACTACCTTACGACG | 3'-33B15NGSP | AGGTCTGCAATCCACTGATGAGG |
| UGT33B16 | 33B16F | CCAATGTGGGGAGACCAGTGG | 33B16R | ACCCTGTTCCTTCTCCCACTC |
|  | 3'-33B16GSP | CTGTAACATTGTCATCGTAAGGG | 3'-33B16NGSP | GAGTGGGAGAAGGAACAGGGT |
|  | 5'-33B16GSP | ATGTCTTTAATATCATCCCTCCAC | 5'-33B16NGSP | GGCGTGATAATGATCAGCTCATG |
| UGT33F5 | 33F5F | ATTATTTCGTAATAATTGACAATA | 33F5R | GCAGCACGTACTCCGTCCAC |
|  | 3'-33F5GSP | CTTACGATATTCTATGGAAATGG | 3'-33F5NGSP | ATGGTGGACGGAGTACGTGCTGC |
| UGT33F6 | 33F6F | AGTACGCTGGGACGGCTGCCC | 33F6R | CATTGAACCATTGATCAGCCAAC |
|  | 3'-33F6GSP | GATGTGCTTTGGAAATGGGACAAG | 3'-33F6NGSP | TGTTGGCTGATCAATGGTTCAATG |
| UGT33F7 | 33F7F | ACAGTCTACACGCAGCATACG | 33F7R | CCACCACACAGCGCGCTCTAG |
|  | 3'-33F7GSP | TTGCCTTACGACGTGCTATGGAAG | 3'-33F7NGSP | GGAGTTAGATACTCTTACCGATG |
| UGT33F8 | 33F8F | ATGCTAACGTTGGCGCATCGG | 33F8R | GGCAGGTCCTCGCAGATGTC |
|  | 3'-33F8GSP | GAAGCTATATCAGCTGGTGTACC | 3'-33F8NGSP | CGAGACATCTGCGAGGACCTGCC |
|  | 5'-33F8GSP | TTCAGTCACTGGTGGAACATCTC | 5'-33F8NGSP | GACCGATGCGCCAACGTTAGCAT |
| UGT33J3 | 33J3F | TTGCAAACACGACCTCACGATGG | 33J3R | CTCAGTCTGTTAATGTTCTTGCG |
|  | 3'-33J3GSP | GGCTATAACTGCAGGAGTTCCTC | 3'-33J3NGSP | CCGCAAGAACATTAACAGACTGAG |
| UGT33T3 | 33T3F | GAACCCGAAGATTTCATTCAATCG | 33T3R | GATCAAGTTCTAAGTACTGTCTC |
|  | 3'-33T3GSP | CGACAGTTATCGTCGCAATATGG | 3'-33T3NGSP | ATCGTGGAGACAGTACTTAGAAC |
| UGT33V1 | 33V1F | TCAAAATGTCACATTTCCTC | 33V1R | TTCTAGTTCGAGGTACTCTG |
|  | 3'-33V1GSP | AAGGAGGTCTACAATCAACAGATG | 3'-33V1NGSP | TGTGATATACGACCAGCCTCAACC |
| UGT33V2 | 33V2F | CCGACTTCAACCAAGCAGGTC | 33V2R | TGGTACAGGATAATTGCCTTC |
|  | 3'-33V2GSP | AGTGATGCTGAACAAGAACTATGC | 3'-33V2NGSP | CTTTGAAGGCAATTATCCTGTACC |
|  | 5'-33V2GSP | GAAATACCACTTGGTGACTGATCG | 5'-33V2NGSP | CGTGACCTGCTTGGTTGAAGTCGG |
| UGT33V3 | 33V3F | CCCTCAGTGTAACGTTGGCAGTCG | 33V3R | CGAGGTACTCTGCCCACGACA |
|  | 3'-33V3GSP | CAGATGAGGCTATATCAGCAGGAG | 3'-33V3NGSP | TGTGTGGTGGACGGAGTATGTGC |
| UGT33V4 | 33V4F | CATGAGCTGATCATTATCACGCC | 33V4R | ACCCTGTTCCTTCTCCCACTC |
|  | 3'-33V4GSP | CTGTAACATTGTCATCGTAAGGG | 3'-33V4NGSP | GAGTGGGAGAAGGAACAGGGT |
|  | 5'-33V4GSP | ATGTCTTTAATATCATCCCTCCAC | 5'-33V4NGSP | GGCGTGATAATGATCAGCTCATG |
| UGT39B4 | 39B4F | AAAATGGACACTTTAAAAATAA | 39B4R | CTATCATGGTGTACGCAAACC |
|  | 3'-39B4GSP | TACCATGACATCAATGAGGAGAC | 3'-39B4NGSP | TTGGTTTGCGTACACCATGATAG |
| UGT40D3 | 40D3F | ACATCATGGAGAGAGCGAAG | 40D3R | CAGCGAGATCCAGGTACATC |
|  | 3'-40D3GSP | TTACGAGTGGACCTTTCGTATTC | 3'-40D3NGSP | GAAGATGTACCTGGATCTCGCTG |
| UGT40D5 | 40D5F | GTATGTATATATAACTTGGCTAG | 40D5R | GAATAGGAGTACTAGGACTGC |
|  | 3'-40D5GSP | GTATACCAGTATTCGCTGACCAG | 3'-40D5NGSP | CTGCAGTCCTAGTACTCCTATTC |
| UGT40F3 | 40F3F | AAGTCATGGCATCCTTGGAGAC | 40F3R | ATTCGGATATGACCAATCACAG |
|  | 3'-40F3GSP | CGCTCTGGATAAAGTTCTTGCTG | 3'-40F3NGSP | ACTAGCTGTGATTGGTCATATCC |
| UGT40F4 | 40F4F | CATTNGGATTGTCAGTTGTGTG | 40F4R | GGTACAGCGGCACCTGTAGCGC |
|  | 3'-40F4GSP | CTCACCACTTACATAAGGACCTG | 3'-40F4NGSP | AGTAGCGTTGAACTTCTGGGTGG |
| UGT40F5 | 40F5F | GCAGCTGTTAAAGTGGACCTCAC | 40F5R | ATACTATGATCAAGCATGGATCG |
|  | 3'-40F5GSP | GTTCTTACAGATATTAGCGTGAT | 3'-40F5NGSP | TTCGATCCATGCTTGATCATAGT |
|  | 5'-40F5GSP | CTGTATACTTCGGAAGTTCTGGG | 5'-40F5NGSP | GTGAGGTCCACTTTAACAGCTGC |
| UGT40L3 | 40L3F | TGCCAATTGTGATGCACGTGAG | 40L3R | GGACAGCTGCATCAAGTTCAGG |
|  | 3'-40L3GSP | GACATGAAGGATGATATACCTCC | 3'-40L3NGSP | ACCTGAACTTGATGCAGCTGTCC |
| UGT40M2 | 40M2F | ATCAGCAGCAATGTTGCTTTTG | 40M2R | TAAAGGTACGTGAAGAGCTGGAG |
|  | 3'-40M2GSP | AAGAGCTATCCTCTCTCTACCATG | 3'-40M2NGSP | GTGCTTCTCACCTACGCTCTCCAG |
|  | 5'-40M2GSP | TCACGCGAGGAACTCATCTCAAG | 5'-40M2NGSP | AACAAAAGCAACATTGCTGCTGAT |
| UGT40M3 | 40M3F | CACTGACATCTTTGCGTTGGTCTG | 40M3R | AGGTACGTGAAGAGCTGGAG |
|  | 3'-40M3GSP | GCACTAATCAAGGAGATACTCCAG | 3'-40M3NGSP | TCTACCATGATCGCTTGACGAAGC |
| UGT40Q2 | 40Q2F | AGTTGTTAAGTGCGCGGCAAATC | 40Q2R | GTTCGCAATCTAAATGGACG |
|  | 3'-40Q2GSP | TGGCATACCGTTATTTGCTGACC | 3'-40Q2NGSP | AGCTCTTCACGTCCCAGTGTATC |
| UGT40R4 | 40R4F | TCATCTCCTAACACGGAGAGGTTG | 40R4R | GTAAAGCAGGAGAGCGCAGG |
|  | 3'-40R4GSP | CCTGTCTTCGGAGATCAGTTCATC | 3'-40R4NGSP | CACTGGGTACAGCATGTAGTGAAC |
| UGT40U2 | 40U2F | CTCAAAGCATGGATTCATTTAC | 40U2R | TGATGAGGACTGCGGCGAGGTC |
|  | 3'-40U2GSP | ATGGCTGATGAGCTCAAGAAAGC | 3'-40U2NGSP | CTACCAGAAGATGTACCTGGACC |
|  | 5'-40U2GSP | GTGCCCATTCTAGAATGTGAACA | 5'-40U2NGSP | AAGTAAATGAATCCATGCTTTGAG |
| UGT42B5 | 42B5F | TCAGTGTACTGCCGGAGGCCACAG | 42B5R | GAGCACAGCAGCTACGTCTAGG |
|  | 3'-42B5GSP | ACTATGGAGTACCAATGGTAGCC | 3'-42B5NGSP | AACCTAGACGTAGCTGCTGTGCT |
| UGT42C2 | 42C2F | GAGTAGACTGAACGTGCGGGCAT | 42C2R | TGGCATACATGTAAGACAAATTG |
|  | 3'-42C2GSP | TTCAGAACTCCAGCTAACTCCGT | 3'-42C2NGSP | ATGCTTGCCCACTAAGTACAGTC |
| UGT43A2 | 43A2F | GGCAACATAATGTGACAGTG | 43A2R | AGTACTGATACAGTGGCATG |
|  | 3'-43A2GSP | GGAATGCTGAGTACATCAGAAGC | 3'-43A2NGSP | ACATGCCACTGTATCAGTACT |
| UGT44A5 | 44A5F | CACACACATTGAAGCTGTATACC | 44A5R | GAGATCTTGGTAGTGCACGATGG |
|  | 3'-44A5GSP | AATCTGCAACTGCCGAGAAACGT | 3'-44A5NGSP | CCATCGTGCACTACCAAGATCTC |
| UGT46A7 | 46A7F | CAAGGAGTAGAAACCATCGACCTG | 46A7R | TTCATGTTGAAGCAATTCATAC |
|  | 3'-46A7GSP | TTTAGTGCTATTTGGCTCTGTT | 3'-46A7NGSP | CATTTCTTAGTAGTAGCTCAATG |
|  | 5'-46A7GSP | TTTCCTGCTGTGGTCCAAGTGC | 5'-46A7NGSP | CCGGCAATAGAGGACGAACACC |
| UGT48D1 | 48D1F | AGAAGATTATCTTGATCGAGATGC | 48D1R | GCGTGATCATCATGATGGTGT |
|  | 3'-48D1GSP | CTTCAAACCCTGCTGGATAACGC | 3'-48D1NGSP | GAACACCATCATGATGATCACGC |
| UGT50A5 | 50A5F | GAATCACAGCAAAATGCATAGGTC | 50A5R | CTTCGAACTTGTCAACAACTCG |
|  | 3'-50A5GSP | GTACTCGTAACATTATACGCTCTT | 3'-50A5NGSP | CCAATTGTTGGACAAGTCAAACG |

**Table S5 Primers used in quantitative real-time PCR of Cytochrome P450 genes**

| No. | Name | Sense primer | Anti-sense primer | Amplicon  (bp) | qRT-PCR  efficiency (%) |
| --- | --- | --- | --- | --- | --- |
| 1 | CYP4G75 | CCGACTTCAAACTGCAAGCT | AATCAACACGCCTTTGCCAA | 94 | 100.09 |
| 2 | CYP4G74 | CTGTCGACCATCCTCCGTAA | CTAAGCGACGTTGGGGATTG | 150 | 97.34 |
| 3 | CYP4L15 | GCATTGGTCAAAAGTTCGCG | TCTGGTTCCTGAGCTGATGG | 94 | 94.92 |
| 4 | CYP4L9 | GCATTTCGTACACGCTCTA | GTTCCTGGTAAGTTGGGTC | 117 | 98.12 |
| 5 | CYP4L7 | ACTGCAGACAATGTTCGGTG | CGCATCGACTCCTTAATGACC | 99 | 97.23 |
| 6 | CYP4M18 | GACCTGTATCCCAACCCGAA | AATTTCTTGGCCCTGCACTG | 109 | 94.87 |
| 7 | CYP4M17 | GTAACCACTCAATTCCCGCC | AACACTTCAGGGTTCGGGAA | 91 | 93.57 |
| 8 | CYP4M15 | GCAGACACGCAGGAAGATTC | TCTAGGAACCGTTCGCTGTT | 96 | 98.89 |
| 9 | CYP4M14 | CGGGCATGAAGTTTGGTGAA | AGTGGAAAGCTGGAGTGAGG | 129 | 97.90 |
| 10 | CYP4S8 | ACAACTCCTCTCGTGGGAAG | ATTCTGAGCTCTTTGGGCCT | 101 | 95.67 |
| 11 | CYP4S9 | TGGTGGTGTACTGATGGGTC | ACGTCATCAGCGAACGAATG | 126 | 94.35 |
| 12 | CYP6AB61 | CACTCAAGGGGCCTCACTTA | CACAAGTCACCGTCAGCAAA | 80 | 98.99 |
| 13 | CYP6AB12 | GGAAAGCCAGTATGACGCAG | AGAGCGAAGAAATCCGACGA | 80 | 101.1 |
| 14 | CYP6AB31 | AGCAGAAGGGAGTGATGGTC | CCAGCCGCGAAGAATACAAA | 127 | 99.98 |
| 15 | CYP6AB14 | TCTTGATGCTGACTCGCTCA | TACAGGCTTCCGGGAACATT | 125 | 95.46 |
| 16 | CYP6AE10 | GGACAATGGTGAAGACTGGC | TGCGACAAACTTGAGTGCTC | 91 | 97.24 |
| 17 | CYP6AE97 | GGATGATGAGCTGCTGGTTG | GCATGAACTCGTCGACTTCC | 149 | 98.45 |
| 18 | CYP6AE47 | GCATGGAGTCGATGCGTATT | TCCAAGGACCGGGTAGAAAC | 121 | 104.7 |
| 19 | CYP6AE74 | GTGGGAGACAGCATCAGGAA | ACCCAGCAGCAAAGAAAACA | 109 | 105.2 |
| 20 | CYP6AE68 | AGTATGTCGAATATGAAGGGTG | CGTAGTCGCCGATGTCTC | 120 | 94.89 |
| 21 | CYP6AE70 | ATCTCACGCCTCTGTTCTC | GTCAAAGTCCTCGTATCAA | 196 | 96.77 |
| 22 | CYP6AN4 | GTTCCCTCTCGTAGTGCAGT | GTGGTGAAGCGAGCCATTAG | 108 | 98.22 |
| 23 | CYP6B31 | AACGTCTGATGTGATTGCGG | CTTCTTCTGGACGTCTGGGT | 120 | 97.34 |
| 24 | CYP6B68 | AGAATACAAAGTGCCCGGGA | CAGGGTTGAATTGTTCGGGG | 119 | 98.47 |
| 25 | CYP6B50 | TGTGAGAGAACTGCATCCCT | GGAGCTGTGCGAACTTTGAA | 83 | 97.89 |
| 26 | CYP18A1 | CTTATCGCCAACCTTCACCG | GTTATGCCGCAGATGACGTT | 92 | 93.99 |
| 27 | CYP18B1 | ATTCAGCAACGCGAACAGTC | ACGGGATCAACATCTGGGAA | 140 | 92.98 |
| 28 | CYP301A1 | GCGGATGGTGCCTATAATCG | TCTTCCAATCAAGCCGCCTA | 112 | 96.84 |
| 29 | CYP301B1 | CCGACAAATAAGGGATGAA | GTCTGCACTGCGTGAATAA | 165 | 94.95 |
| 30 | CYP302A1 | CCTCTCGGATGCCACAGTAT | TATACATGACCGTGGCCCAA | 134 | 100.2 |
| 31 | CYP304F1 | ACTGACGGTTACTTCTGGCA | CATTTCAGCGGGGTACTTCG | 162 | 102.6 |
| 32 | CYP305B1 | TATCCCGTAGTCTGCATGGC | CCACAACTCTGGGTCGAAGT | 129 | 99.12 |
| 33 | CYP306A1 | AGATCCAAGAAGAGGGGTGC | TCTGAGCTTGACCACGTGTT | 126 | 98.72 |
| 34 | CYP307A2 | GGCTCGCACCATTCTACAAG | AGTCCTTTTCTGGTCCCTCG | 132 | 99.01 |
| 35 | CYP314A1 | CCTACGTTGGTACTGGTGGT | TACTCCAGAAAAGCCACCGT | 145 | 97.56 |
| 36 | CYP315A1 | AGCTGGTGATACGACGTCTT | GCGCAACGGGATATAACCTC | 143 | 99.21 |
| 37 | CYP321A16 | GCCGTCGGTATAGGTCAGTT | CAATGCTTTCCACTCCTCGG | 118 | 100.6 |
| 38 | CYP321A9 | CAGAAAGGAACCAGCCATCG | CCTTCGACGCTTTCCATACC | 135 | 103.4 |
| 39 | CYP321A8 | AAACAACCCCAAGACGCATG | CCAATGCCAAAGATAGCCCC | 90 | 105.6 |
| 40 | CYP321B1 | TGGAGCCTACAGACGAGTTG | TCCAAAAGTTCCGGCAGTTG | 86 | 91.99 |
| 41 | CYP321B4 | TTCACGACCTGGGACTGTAC | TGGACCGTTGAGCATTAGGA | 143 | 102.7 |
| 42 | CYP324A1 | GTCCTGGTCCAGCATCTTCT | CCTCTCTGTTCCACCACTGT | 120 | 95.67 |
| 43 | CYP332A1 | CTGAAAGATCTTGCCTCGCC | CCTCTCTGTTCCACCACTGT | 110 | 94.89 |
| 44 | CYP333A12 | TGCGAGCGTGTTTGAAAGAA | TGGGGCTATTACATCCACCC | 125 | 93.27 |
| 45 | CYP333B4 | CACGTTGCTCACACTACTGG | TGTCCTGAGCCACTTCTTCA | 139 | 90.88 |
| 46 | CYP333B40 | GCAGCAACCAAAACAGAAAACT | TTACCCAATGTTCCTCCGGG | 110 | 95.44 |
| 47 | CYP337B5 | CCATCTGCGACAAAACCCTT | GGCTCCAGAGTCTTCTTCGT | 144 | 101.7 |
| 48 | CYP338A1 | AGCCACATTCCGACGTAGAA | TTCTCTTGCACCTCGGGATT | 130 | 100.7 |
| 49 | CYP340AA1 | TCATTGTGTCGTTCTGTGCG | GATGAGCGTGCCCCAATATC | 108 | 99.78 |
| 50 | CYP340AB1 | AACTACACACTGAGAGCGGG | AAAACGCATTGGGATTGGCT | 145 | 97.45 |
| 51 | CYP340K4 | GCACATTAGGTATTGAAGCCGT | TATGACGTCACTGAGGAGCC | 125 | 98.32 |
| 52 | CYP340L1 | TGCGAGAAACCTTCAAACCG | ACTCTCTTGCATCTGGTCCC | 122 | 99.70 |
| 53 | CYP341A11 | CCCTCCCGTGCCACTTATTA | GTCGGGGTCAAATTTCTCGG | 157 | 98.56 |
| 54 | CYP341B26 | GTGTGTGAAACTGCTCTCGG | ACCATACGCTCGGCTATCAA | 107 | 93.67 |
| 55 | CYP341B27 | AGCCTTCCTCGGTACACAAA | ACCCTGGGTCGATTTGTTCT | 114 | 94.45 |
| 56 | CYP354A14 | GCGAAACTGGCTATGGTTGA | TCTACACGAACCCACAGACC | 131 | 96.45 |
| 57 | CYP366A1 | CTGATGGTGTACAGGGGTGT | GCAGAACAGCAACGCATTTC | 100 | 98.65 |
| 58 | CYP367A1 | CAGCCAGTTGGTTGGTTTG | GAGTGGAAGTGCGGTTGGA | 86 | 99.07 |
| 59 | CYP367B1 | AAATTCTGGCTCGGTCCTGA | TTGTATTGCGGCCCTTTCAC | 104 | 98.79 |
| 60 | CYP9A10 | GGCGTTGCCCTTGACAGAC | ACGGTGGATTGGATAAACTGG | 126 | 97.67 |
| 61 | CYP9A27 | ATGACGATAAACCCAATG | CAAAGCCAGCGATAAAGA | 141 | 95.78 |
| 62 | CYP9A98 | CTACCAGCATCTGCGTCAC | TTAGCCTACACCTTAACCAAT | 122 | 94.09 |
| 63 | CYP9A11 | ATCTTACAACTCGCTACGC | GCTGCTGTCCTACCCATTA | 112 | 94.56 |
| 64 | CYP9A97 | TTTGCGCTTTGTGAGATAA | CTAAGTCCTTGCCCTGAAC | 159 | 93.78 |
| 65 | CYP9A9 | ACTACCCGCTACACAAACGA | CTCTTTCCCCAGTGCGTAGA | 102 | 92.89 |
| 66 | CYP339A1 | CAGATTTACGGTGGATTGT | CTTCATTGATGCCGCTTAT | 115 | 91.45 |
| 67 | CYP324A6 | GATGAACTCGTGGAAGCAG | ACCAGCCCATGATAGCGTA | 127 | 98.56 |
| 68 | CYP428A1 | AGCCATGAGACTCCATCCAG | TTTGCTTGTGACACCGTGAG | 124 | 104.23 |
|  | GAPDH | CTGAGGAACAGGTCGTGTCATC | GATCGATAACGCGGTTGGAGTA | 150 | 99.67 |

**Table S6 Primers used in quantitative real-time PCR of UGT genes**

| **No.** | **Name** | **Sense primer** | **Anti-sense primer** | **Amplicon**  **(bp)** | **qRT-PCR**  **efficiency (%)** |
| --- | --- | --- | --- | --- | --- |
| 1 | UGT33B14 | ACCACACGCTGGTCAACATA | GCGCTTTCACTACATGCGAC | 141 | 98.34 |
| 2 | UGT33B15 | TGGGAGGACTGCACCAAAAG | AGGTAGCAACGATGGCAACA | 125 | 98.23 |
| 3 | UGT33B16 | GCAGTCGCTTTGTTCTTGCT | GAACGCAGGATCTGGTGTGA | 171 | 96.45 |
| 4 | UGT33F5 | AGATCCCACGCAATTCCCAG | TGGCAGCCATTTTGCAATCC | 142 | 93.21 |
| 5 | UGT33F6 | GAACTGCCAGGACGCTCTAA | CAGCCAACATCGGTACTCCA | 166 | 90.88 |
| 6 | UGT33F7 | ACTCGTGGAAGCTTGTGTGA | TCTCTCGAGTGAAGGCAGGA | 155 | 89.77 |
| 7 | UGT33F8 | TGAAGGGATTCGACCAGTGC | AGTGGGATCCACGTTTGTACC | 154 | 101.2 |
| 8 | UGT33J3 | CAGTGCCACCGAGTGTTGTA | AAGGCCTTCACAAGGGTCTG | 181 | 90.78 |
| 9 | UGT33T3 | ACCACTCCTCGTACTCTCACA | CAGTTTGTCCCAGACCGTCA | 166 | 106.1 |
| 10 | UGT33V1 | GACCAGCCTCAACCTCCA | GGTACTCTGCCCACGACA | 115 | 103.2 |
| 11 | UGT33V2 | GTGCCATCGATCAGTCACCA | TTCTGTGAGATTCGCAGGGG | 132 | 98.67 |
| 12 | UGT33V3 | GCCTCACAAGCCTCTACCAA | TCCACTTCCATAGCACGTCG | 173 | 99.04 |
| 13 | UGT33V4 | CAAGGAGGTCTACAATCA | TTTCTTCAGTAAGCGTCT | 151 | 99.45 |
| 14 | UGT39B4 | TTTTGGACGTCAGAGACCCG | GGTTTCGGCAAATCCGGAAG | 145 | 101.3 |
| 15 | UGT40D3 | GAGAACGTTGGCTCGCTAGT | CCAGATGAGTGGGCAGTTGT | 180 | 101.5 |
| 16 | UGT40D5 | CAGTGCTGAGGCTTCCAAGA | GGAGCTTGGGAGGAGCATTT | 155 | 104.2 |
| 17 | UGT40F3 | TTGGCACTGTTCGTCACTCA | TCCTTTGTGTTGGGCTTGGT | 129 | 107.2 |
| 18 | UGT40F4 | GCAGGGTCTGTTGGAGGT | TCAAATTCGGATGGGCTA | 140 | 94.76 |
| 19 | UGT40F5 | GTCGACGTATCTGCAAACGC | TTGGGTTGCCAAGAGTGTGT | 145 | 93.89 |
| 20 | UGT40L3 | GATTAACGCCCACCCTTCCA | CCGACATGCCATCACTCTGT | 185 | 92.45 |
| 21 | UGT40M2 | GTACCCATTGAAGAACCCGC | TTGCAGCTTCTTGGCCAAAT | 167 | 90.89 |
| 22 | UGT40M3 | CGGAAAGCCGGTCATAGGAA | GGCTTTGTTGGCAAACCGAT | 169 | 91.34 |
| 23 | UGT40Q2 | TGGCGTCGGTAATCGACAAA | TGTGTAAGCGACCTGTTTGC | 136 | 92.56 |
| 24 | UGT40R4 | GAATTGCCGAATGTGCCGAA | ACAGGTATGGCAATGGTCGG | 158 | 90.99 |
| 25 | UGT40U2 | AAGAGGTCAGGGTGAGCCTA | GCTTGTTGTGGTGCCCATTC | 127 | 94.50 |
| 26 | UGT42B5 | GGTTCCTTGCTCATAGCGGT | GCGACACCAAGAGCACAATC | 139 | 98.77 |
| 27 | UGT42C2 | GACTTTACCGGGAAACCCGA | TCCGTGGTTCCTAACATGCC | 120 | 99.03 |
| 28 | UGT43A2 | TTCTAATAAACTTCGGGATG | TTCCACCAACTTCAACAA | 198 | 97.89 |
| 29 | UGT44A5 | CGTGAACCTCGGCTCTAC | GTGGGAACCATCTCATAGTCAT | 161 | 99.07 |
| 30 | UGT46A7 | AACGCTGACCGGCAATATCT | AGCAGAAATGGCTTCCGTCA | 124 | 89.56 |
| 31 | UGT48D1 | TAACGACGTTCACGAGGGTG | AAACGGGAGGAGCTTGGTTC | 200 | 98.04 |
| 32 | UGT50A5 | CATCATTCCCTTCCACGCCT | TAGGGTCTCGGGTAGGACAC | 153 | 103.5 |


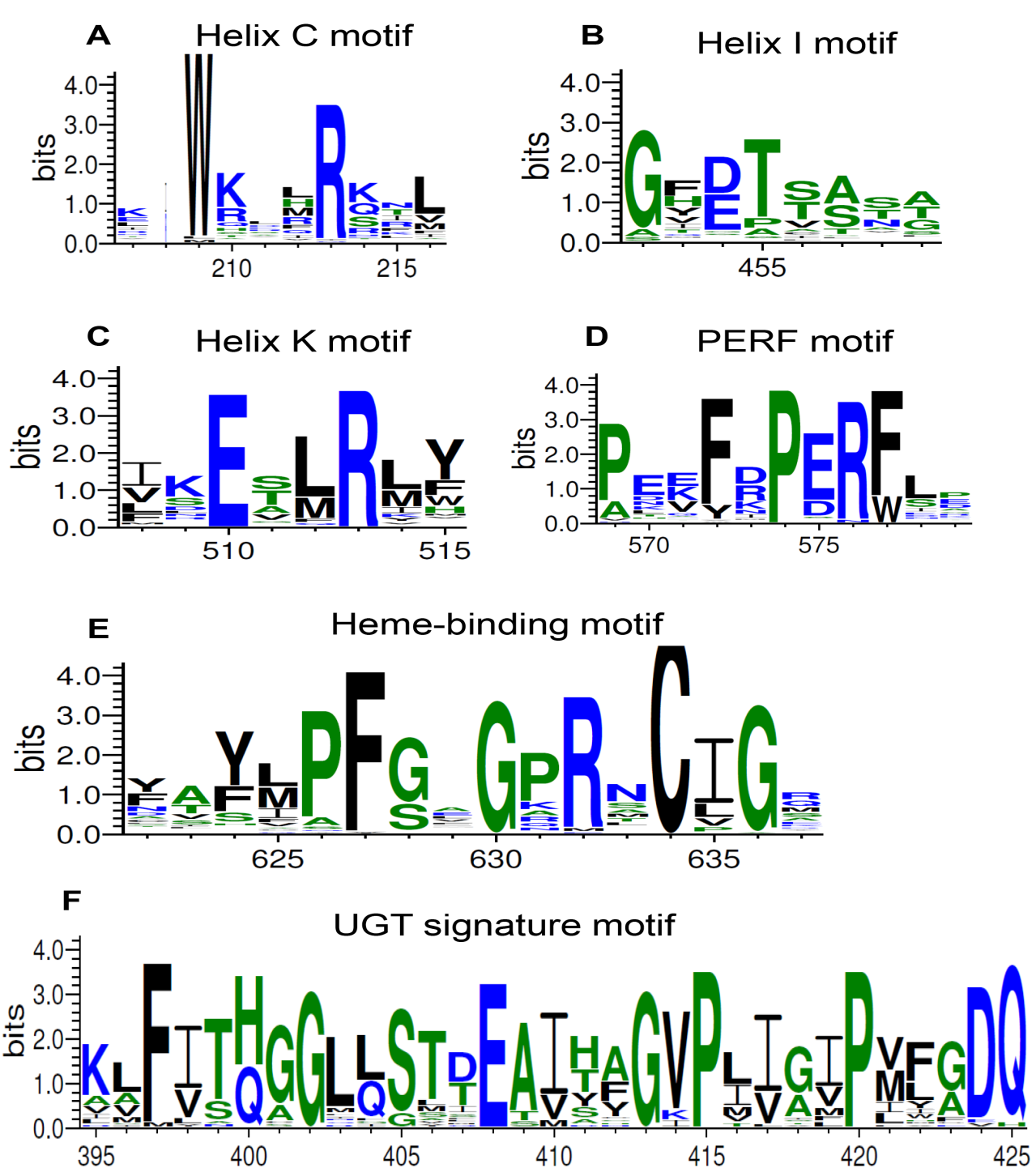


**Figure S1 The conserved motifs of the P450s and UGTs from *S. exigua*.** The horizontal axis represents the position of amino acids, and the vertical axis denotes the information content in bits. Sixty seven P450 protein sequences and thirty two UGT protein sequences from *S.exigua* were aligned by ClustalX 2.0.12, and the conserved motifs were generated using WebLogo 3.4. (A)The helix C motif (WxxxR) sequence logo. (B) The helix I motif (Gx[ED]T[TS]) sequence logo. (C) The helix K motif (ExLR) sequence logo. (D) The PERF motif (PxxFxP[ED]R) sequence logo. (E) The heme-binding motif (PFxxGxRxCx[GA]) sequence logo. (F) The UGT signature motif: [FVA]-[LIVMF]-[TS]-[HQ]-[SGAC]-G-x(2)-[STG]-x(2)-[DE]-x(6)-P-[LIVMFA]-[LIVMFA]-x(2)-P-[LMVFIQ]-x(2)-[DE]-Q.


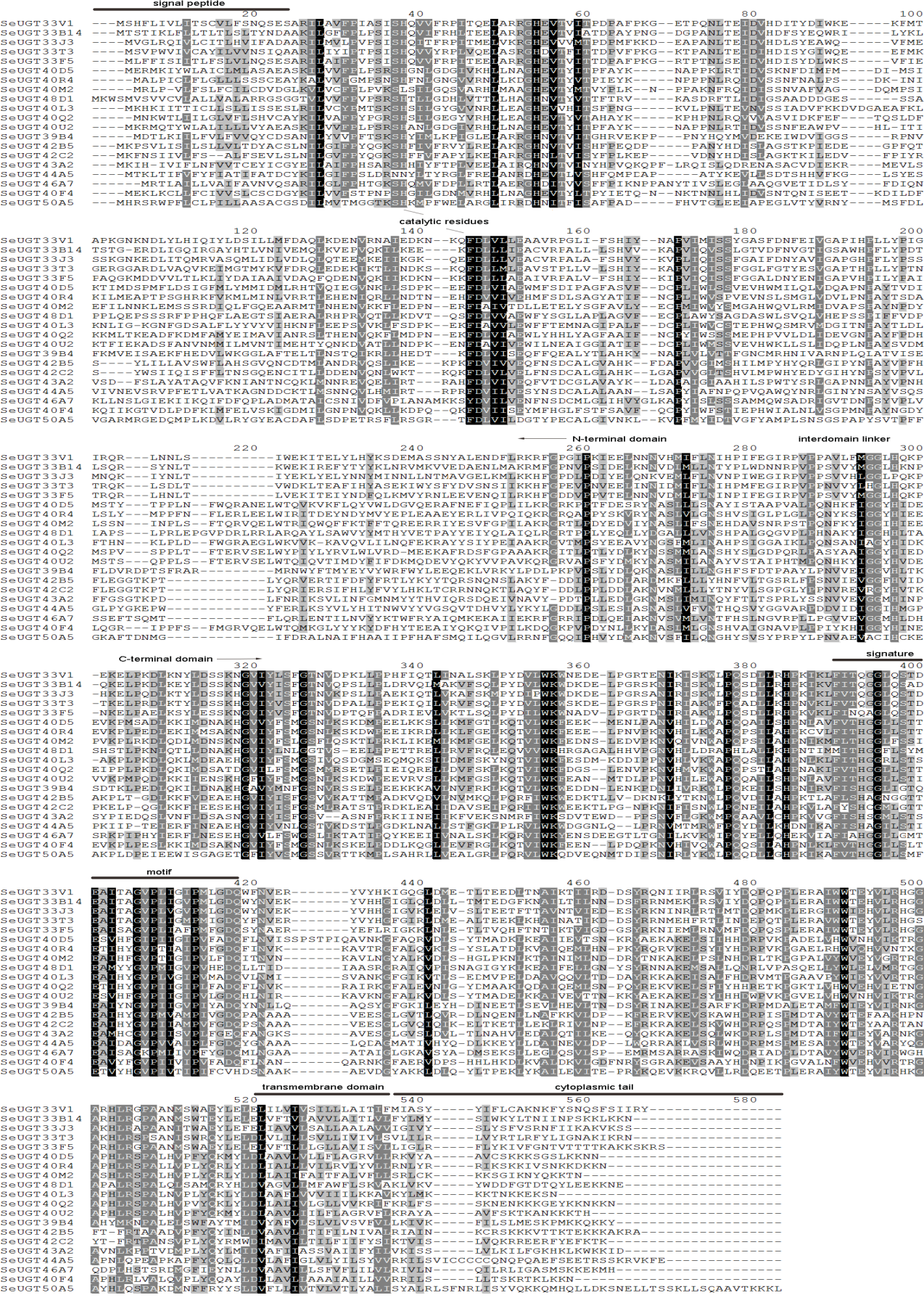


**Figure S2 Amino acid multiple alignments of the 20 representatives of *S. exigua* UGTs.** Multiple sequence alignment was performed with ClustalX and displayed by GENEDOC. Signal peptide in N–terminal was predicted by SignalP 3.0. Black and gray indicate identical and similar amino acids. Signature motif, transmembrane domain and cytoplasmic tail are also showed in the sequence alignment.
